# Persistent lineage plasticity driving lung cancer development and progression

**DOI:** 10.1002/ctm2.70458

**Published:** 2025-08-22

**Authors:** Fanchen Meng, Jianyu Li, Zhijun Xia, Qinglin Wang, Qinhong Sun, Siwei Wang, Lin Xu, Rong Yin

**Affiliations:** ^1^ Department of Thoracic Surgery, Jiangsu Key Laboratory of Innovative Cancer Diagnosis & Therapeutics Jiangsu Cancer Hospital & Nanjing Medical University Affiliated Cancer Hospital & Jiangsu Institute of Cancer Research Nanjing China; ^2^ Department of Science and Technology Jiangsu Cancer Hospital & Nanjing Medical University Affiliated Cancer Hospital & Jiangsu Institute of Cancer Research Nanjing China; ^3^ Collaborative Innovation Center for Cancer Personalized Medicine Nanjing Medical University Nanjing China; ^4^ Biobank of Lung Cancer, Jiangsu Biobank of Clinical Resources Nanjing China

**Keywords:** histopathologic transition, lineage imbalance, lung cancer, lung development

## Abstract

**Background:**

Lung cancer, a leading cause of cancer death, displays profound histologic and molecular heterogeneity across adenocarcinoma, squamous, and small‐cell types. Clinically, tumours can shift between these states, reflecting lineage plasticity—the reprogramming of differentiated cells to alternate identities. Pre‐existing genomic/epigenomic diversity and microenvironmental cues supply the substrates and pressures for plasticity from disease onset. This review anchors plasticity within normal lung development to clarify how fate programs are co‐opted to drive progression, immune escape, therapy resistance, and invasion.

**Main text:**

Focusing on the intricate interplay between lineage dysregulation and tumour progression in lung cancer, this review integrates insights from lung tissue development to explore the pivotal molecules and mechanisms driving lineage plasticity, alterations and migration during lung carcinogenesis and progression. Recent research findings on lung cancer lineage plasticity are synthesised, shedding light on the role of transcriptional and epigenetic regulators in disrupting tumour lineages. Particular emphasis is placed on how tumour microenvironmental factors, such as hypoxia, stromal cells and immune cells, reshape tumour cellular profiles by modulating the epigenomic landscape. Furthermore, this review specifically discusses the impact of epidermal growth factor receptor (*EGFR)* and *KRAS* mutations on lung cancer progression and the consequent immune escape mechanisms they engender. Importantly, we highlight that lineage regulation persists throughout tumour development, from the early onset of lung adenocarcinoma (LUAD) to its progression through late‐stage dedifferentiation and metastasis. We evaluate the implications of these factors on treatment resistance in lung cancer and focus on innovative therapeutic strategies targeting lineage plasticity.

**Conclusions:**

Lineage plasticity spans the entire course of lung cancer, from early tumorigenesis through metastasis to treatment resistance. Lineage transitions that occur during tumour progression arise from specific combinations of genomic and epigenetic alterations and are further shaped by microenvironmental forces such as hypoxia, stromal remodeling, and immune pressure. By summarising current research advancements, we aim to provide new insights for future lung cancer research and to promote the development of more effective therapeutic interventions.

**Key points:**

- Lineage plasticity runs through the entire process of lung cancer progression and drug resistance, and drives early tumorigenesis via lineage imbalance.
- Certain driver mutations have lineage‐restricted tumorigenic potential, requiring lineage reprogramming for tumor initiation.
- Lineage transitions in lung cancer require specific genomic and epigenetic alterations.
- Lineage plasticity insights provide a mechanistic framework linking lung cancer origin, evolution, and therapeutic vulnerabilities.

## Graphical abstract

Lineage imbalance among tumour‐initiating cell types persists after oncogenic transformation and, in the context of clinical intervention and the accumulation of multiple genomic alterations, ultimately culminates in histologic transformation.

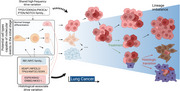

## 1 INTRODUCTION

Lung cancer, one of the most lethal malignancies globally, is characterised by its pronounced heterogeneity and complexity.^1^, ^2^ The predominant histological subtypes include LUAD, lung squamous carcinoma (LUSC) and small cell lung cancer (SCLC). Each histologic type exhibits distinct genomic landscapes and molecular functionalities^3^, ^4^ However, during clinical diagnosis and treatment, tumour cells often undergo interconversion among these pathological types, contributing to tumour progression, immune evasion, drug resistance and invasion.

Recent research has elucidated that lung cancer cells possess remarkable lineage plasticity, enabling them to transition from one histological state to another through alterations in transcription factors (TFs) and signalling pathways under specific conditions.^5^, ^6^ This plasticity is not only evident during histological type conversion but also plays a critical role in tumour initiation and progression. The emergence of lineage plasticity is now recognised as a hallmark of cancer. Tumour cells acquire new molecular characteristics through processes of dedifferentiation and transdifferentiation, thereby altering their identities and adopting features that enhance immune evasion and invasiveness.^7^, ^8^, ^9^

Pre‐existing genomic and epigenomic heterogeneity creates diverse cellular states that can be reprogrammed, thereby seeding lineage plasticity, which in turn generates further heterogeneity.^10^, ^11^ Studies by Finlay et al.^10^ and Zhang et al.^11^ show that such pre‐existing genomic diversity – for example, PTEN loss superimposed on Rb1/Trp53 deficiency or divergent transcriptional programs in olfactory neuroblastoma – produces multiple subclones with distinct epigenetic landscapes. These heterogeneous subclones display differential sensitivity to lineage‐specifying cues and therefore provide a substrate from which highly plastic subpopulations emerge. In other words, heterogeneity offers varied epigenomic landscapes that can be reprogrammed, thereby ‘seeding’ lineage plasticity. Conversely, once plastic clones arise, they further diversify the tumour, amplifying heterogeneity. Advances in experimental biology and bioinformatics have significantly deepened our understanding of lung cancer pathogenesis and progression. Single‐cell RNA sequencing (scRNA‐seq), lineage tracing and clonal bar‐coding reveal that heterogeneous lung tumours rarely comprise fully differentiated, mutually exclusive lineages. Instead, they contain a continuum of transcriptional states ranging from mature epithelial identities to cells that co‐express developmental or ‘injury‐response’ programs.^5^, ^12^ These intermediate cells are able to self‐renew and seed recurrent, multi‐lineage lesions after therapy.^5^, ^12^ They can repopulate both AT2‐ and basal‐like branches in mouse models, and they expand under stress, thereby fuelling additional phenotypic diversity.^6^, ^13^ Against a backdrop in which many lung epithelial cells retain latent differentiation potential, increasing clonal and transcriptional diversity raises the likelihood that at least one subpopulation will preserve – or re‐acquire – progenitor‐like plasticity. Thus, greater cellular diversity is frequently accompanied by the persistence or re‐emergence of progenitor‐like subpopulations, forming the mechanistic bridge that links diversity to therapy resistance and metastatic competence.^5^, ^6^

Comprehensive investigations into the plasticity of lung cancer lineages and the regulatory mechanisms governing these processes are crucial. Such studies not only elucidate the fundamental principles of tumour biology but also lay the theoretical and technical groundwork for novel anticancer therapies. This review concentrates on the dynamic lineage alterations from the pre‐oncogenic activation phase through to the adaptive transformations and progression post‐tumour formation. By dissecting the molecular and genomic plasticity throughout these stages, our aim is to emphasise that the process of lineage plasticity does not occur only in the advanced stage or the treatment‐resistant stage of lung cancer, but begins to play a role from the very beginning of tumour formation. In other words, lung cancer is driven and influenced by lineage plasticity from the very onset of its origin, and this perspective highlights the key regulatory factors that will be instrumental in devising effective lung cancer control strategies.

## 2 LINEAGE PLASTICITY IN THE LUNG: FROM EMBRYONIC DEVELOPMENT TO CANCER EVOLUTION

Lineage plasticity refers to the ability of differentiated cells to change their fate – transitioning from one lineage to another – and is fundamental to both development and regeneration. Early evidence of this phenomenon dates back to Hans Driesch's experiments on sea urchin embryos, which demonstrated that, contrary to Roux's notion of fixed cell fate after the first cleavage, blastomeres at the 2–4 cell stage retain the potential to regenerate an entire organism.^14^, ^15^ Over time, epigenetic silencing of stem‐ and lineage‐related genes restricts this plasticity, producing mature cell types that, in adults, are considered terminally differentiated; a small pool of stem or progenitor cells persists solely to replace lost or damaged cells.^16^, ^17^ Although quiescent under homeostatic conditions, mature cells can exhibit robust plasticity in response to extreme stress. For example, following injury, biliary epithelial cells in the liver can dedifferentiate into progenitor‐like cells and regenerate hepatocytes (a process that reverses once the insult is removed).^18^ Similarly, in the adult mouse pancreas, near‐total ablation of insulin‐producing β‐cells triggers spontaneous reprogramming of glucagon‐producing α‐cells (and, in younger animals, somatostatin‐producing δ‐cells) into β‐cells, illustrating age‐dependent differences in plasticity mechanisms.^19^ While plasticity underlies repair in normal tissues, its dysregulation can fuel malignancy: cancer cells often hijack these same mechanisms – through genomic instability and rewiring of fate‐determining networks – to drive tumour initiation, progression, metastasis and therapeutic resistance.^20^ In the lungs, therefore, understanding how normal developmental plasticity becomes aberrant in malignancy is essential. The next sections will first examine lung lineage development and then describe the characteristic changes induced by lineage plasticity during lung cancer evolution.

### 2.1 Embryonic origin and early lung development

The astonishing lineage plasticity shown by lung cancer cells stems from complex lineage development during lung development, and this plasticity provides an advantage for its aggressiveness and treatment resistance. Human lung development commences around the fourth week of gestation from the ventral portion of the foregut endoderm, progressing through five distinct stages: the embryonic stage (4–7 weeks), the pseudoglandular stage (5–17 weeks), the canalicular stage (16–26 weeks), the saccular stage (26–38 weeks) and the alveolar stage (36 weeks to 3 years).^21^, ^22^ During the embryonic period, the primary lung buds on the right and left sides gradually branch to form lobular structures. This branching continues with the formation of airway trees during the pseudoglandular stage and further airway development and enlargement during the canalicular stage.^23^, ^24^ Throughout these stages, the primary lung buds generate both proximal airways and distal alveoli via an extensive branching process, collectively giving rise to lung epithelial cells.^25^

The advent and widespread use of second‐generation sequencing technology, particularly single‐cell sequencing, have revolutionised the study of lung lineage molecular characterisation and microscopic lung structure. Recent studies have identified several subclasses of lung epithelial cells based on their distinct characteristics. These include basal cells (KRT17+), ciliated cells (FOXJ1+), club and secretory cells (SCGB1A1+) and the less common ionocytes (ASCL3+), NE cells (ASCL1+) and tuft cells (GNAT3+).^26^, ^27^ Alveolar cells are categorised into alveolar type 1 (AT1) cells (AGER1+ ETV5+), alveolar type 2 (AT2) cells (SFTPB+ SFTPC+), double‐positive cells (SCGB1A1+ SFTPC+) and alveolar interstitial cells, which are found between the AT1 and AT2 cells and exhibit gene expression signatures of both major alveolar cell types.^28^

**FIGURE 1:**
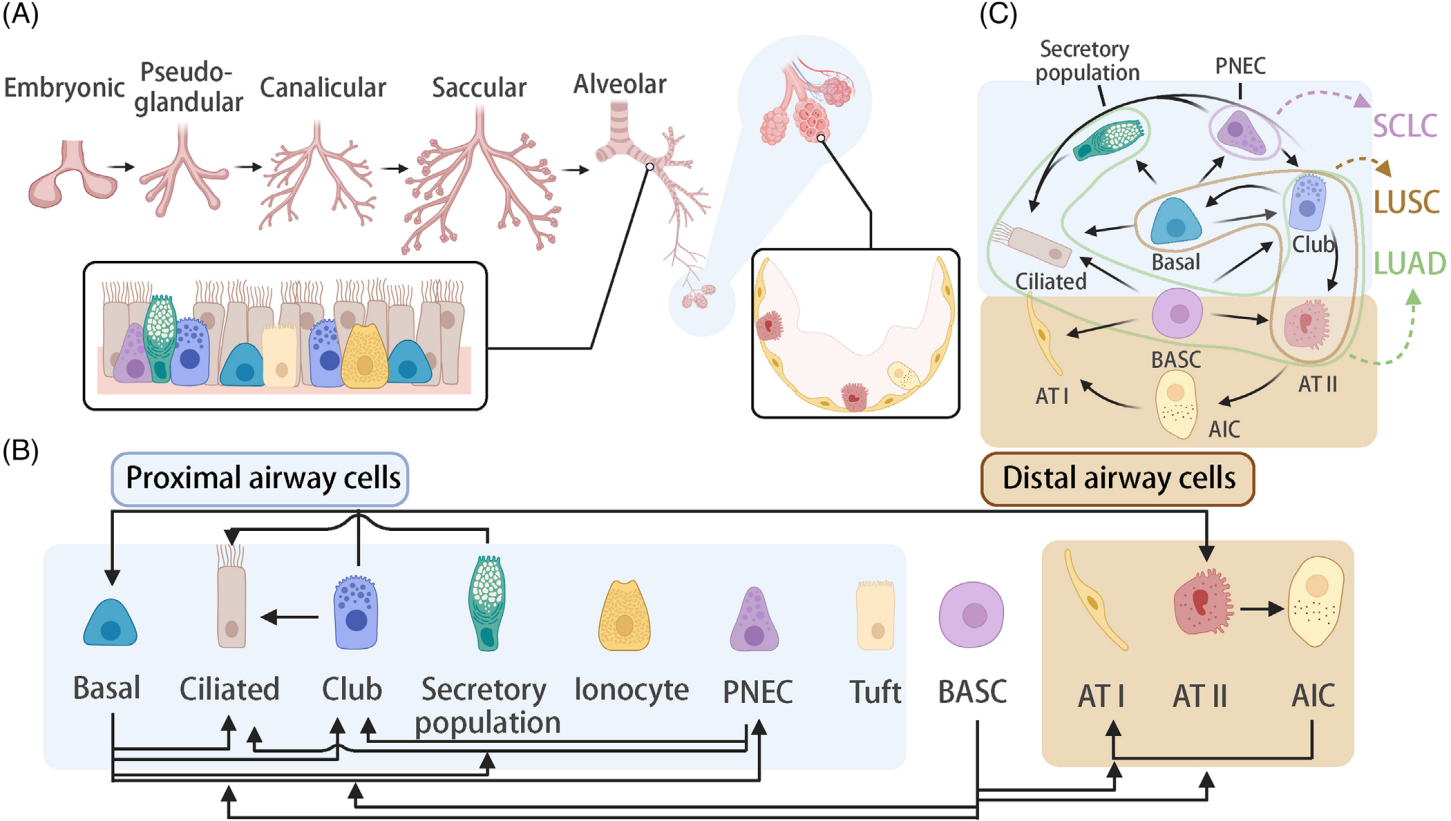
Human lung development. (A) Different stages of lung development and the cellular composition of the proximal and distal airways. (B) The differentiation relationships between different types of lung epithelial cells. (C) Potential progenitor cells of different types of lung cancer. Light blue and brown backgrounds indicate cell types from the proximal and distal airways, respectively, and dotted lines indicate the transformation relationship between the two cell types. *Abbreviations*: PNEC, pulmonary neuroendocrine cell; BASC, bronchioalveolar stem cell. Created in https://BioRender.com.

In the lung, various cell types serve as progenitors in distinct regions. Basal cells differentiate into secretory and ciliated cells within the trachea and main bronchi; club cells can transform into ciliated cells in the finer bronchial epithelium.^29^ In the distal airways, AT1 and AT2 cells emerge directly from bipotent progenitor cells – bronchioalveolar stem cells (BASCs) – during embryogenesis.^29^, ^30^ Furthermore, single‐cell transcriptome analysis has uncovered multiple intermediate maturation stages in this process. In the postnatal lung, AT2 cells acquire progenitor‐like properties, allowing them to further generate AT1 cells^31^ (Figure 1A,B). Most of these characteristics can be verified in mouse models.

Although mice are extensively used as in vivo models for lung development, their lung structure differs significantly from that of humans.^5^, ^26^ Although both human and mouse lungs undergo the embryonic, pseudoglandular, canalicular, saccular and alveolar stages during embryonic development, the exact timelines and morphological processes differ significantly. Compared with human lungs, mouse lung development occurs much faster, with alveolar formation primarily happening post‐birth, starting around postnatal day 5 (P5) and continuing until P36. Therefore, the mouse lung is still relatively filled with fluid at birth, and the alveolar network is incomplete, whereas the human lung has a more mature structure at birth.^32^ The anatomical structure and cellular composition of the proximal and distal airway regions differ markedly between human and mouse lungs. Structurally, the human lung consists of five lobes (three right lung lobes and two left lung lobes) and typically has 17–23 generations of airways, with each lobe extensively subdivided by bronchial branches, including cartilage and a rich submucosal glandular layer.^33^ In contrast, the mouse lung has a simpler lobar organisation (four right lobes and one left lobe), with fewer branching generations (about 13–17 generations of airways). The cartilage in the large bronchi is limited, and the extensive bronchopulmonary segmentation is absent.^34^ Additionally, a prominent feature of human lung morphology is the presence of respiratory bronchioles (RBs), which are intermediate airways connecting the terminal bronchioles and alveolar ducts.^28^ Mice completely lack these RBs; their terminal bronchioles transition abruptly into alveolar ducts at the bronchioalveolar junction.^35^ There are significant interspecies differences in the cellular composition along the airway epithelium. The proximal human bronchus contains a high amount of pseudostratified columnar epithelium, composed of basal cells, club cells, ciliated cells, mucous cells, serous cells, intermediate cells and neuroendocrine (NE) cells, reflecting the complexity of adaptation to prolonged environmental exposure.^36^ The proximal mouse airways are simpler, with low‐columnar epithelium primarily composed of club cells and ciliated cells, lacking basal cells and containing rare mucous cells.^34^ Importantly, the human lung has unique populations of RB‐specific cells, such as AT0 cells and respiratory airway secretory (RAS/TRB‐SC) cells.^28^ These RAS cells are critical unidirectional progenitors of AT2 cells, vital for maintaining and regenerating the alveolar niche^26^ (Figure 1B). The same progenitor populations are not observed in mice, resulting in differences between the two species in airway regeneration and repair mechanisms.

These differences in structural complexity, cellular composition and developmental pathways are pivotal not only for understanding basic lung biology but also for addressing how these species‐specific factors influence lung diseases and cancer. The different timing of differentiation and cellular lineage in human and mouse lungs plays a critical role in shaping their responses to injury, infection and tumourigenesis. For instance, the human lung's intricate structure, with its distinct RBs and cell populations, allows for a more robust response to environmental damage and disease. In contrast, the mouse lung, with its simpler structure and fewer cell types, offers fewer opportunities to study complex human lung pathologies such as chronic obstructive pulmonary disease or idiopathic pulmonary fibrosis.

These developmental differences also extend to the molecular level, with distinct signalling pathways regulating lung formation. In the mouse lung, SOX9 is expressed in distal epithelial cells during the pseudoglandular stage, and as cells differentiate into the proximal airway lineage, SOX9 is down‐regulated and SOX2 is up‐regulated.^23^ In contrast, during human lung development, distal epithelial cells co‐express SOX9 and SOX2 continuously. As human cells move away from the distal tip, SOX9 expression decreases, while SOX2 remains strong, facilitating more complex branching and forming structures like RBs.^37^ The differences in immune system maturation between species further underscore the limitations of mouse models in accurately representing human disease. In mouse lungs, macrophages primarily maintain themselves via self‐renewal under homeostatic conditions, with only limited replenishment from bone marrow‐derived monocytes under severe inflammation or aging. In contrast, human alveolar macrophages, even in the absence of inflammation, are continuously replenished by peripheral blood monocytes.^38^ This dynamic replenishment in humans ensures a more adaptable immune response to infections, chronic diseases and environmental exposures. These interspecies differences are crucial when interpreting immune‐related data from mouse models, particularly when considering immune checkpoint inhibitors and other therapeutic approaches in cancer treatment.

Moreover, substantial differences between human lung cancer and mouse lung cancer models are evident, which limits the potential for direct clinical translation. Histologically, genetically engineered mouse models (GEMMs) are typically driven by oncogenic mutations (such as *KRAS^G12D* or *EGFR^L858R*), primarily resulting in adenocarcinoma‐like lesions.^39^, ^40^ These mouse models typically lack the histological heterogeneity found in human lung cancer, which often includes mixed subtypes, squamous differentiation and various metastatic behaviours. Additionally, human lung cancer features a complex genomic landscape characterised by multiple concurrent mutations and chromosomal alterations, which are rarely fully replicated in mouse models. This gap between mouse models and human conditions is particularly evident when studying the tumour microenvironment and immune interactions, as mouse models typically develop in immune‐naïve hosts under specific pathogen‐free conditions.^21^, ^41^, ^42^ As a result, tumour–immune interactions differ greatly between mouse models and human disease, which can impact the evaluation of therapeutic efficacy. Furthermore, mouse models rarely capture the full spectrum of acquired resistance mechanisms observed clinically, such as secondary *EGFR* mutations (e.g., *T790M*, *C797S*) or *MET* amplification, which further complicates preclinical therapeutic evaluations.^39^, ^43^

Recognising these key interspecies differences is crucial for developing more accurate, predictive and clinically relevant models for understanding and treating human lung diseases. While mouse lung cancer models provide valuable mechanistic insights, their limitations in faithfully representing human lung cancer biology and therapeutic responses must be carefully considered. To bridge this gap, human‐specific organoid cultures, humanised mouse models and lung‐on‐a‐chip technologies are emerging as complementary methods that capture human‐specific biological aspects not present in traditional mouse models. These advanced models offer a promising avenue for improving the translational potential of preclinical research.

In lung cancer research, high‐throughput sequencing technologies such as scRNA‐seq, spatial transcriptomics and single‐cell ATAC‐seq have provided unprecedented insights into cellular heterogeneity, molecular trajectories and microscopic lung architecture.^44^, ^45^ These technologies have elucidated the temporal and spatial dimensions of tumour development, advancing our understanding of the molecular characteristics of lung lineage. Computational tools like SCENIC and Waddington‐OT offer predictive frameworks for understanding how specific cell types form during development by modelling regulatory networks and predicting cell fate decisions.^46^, ^47^ Comparative studies between humans and model organisms (e.g., mice) are invaluable in highlighting the conserved and unique aspects of lung development. Multiple GEMMs, CRISPR/Cas9‐based lineage tracing and single‐cell sequencing have enabled researchers to study tumour progression in native microenvironments.^48^, ^49^ Complementary techniques such as smiFISH have further enhanced our understanding of the lineage integrity of lung cancers during their early initiation and progression by pinpointing the spatial distribution of RNA transcripts within lung tissue. Understanding this diverse developmental process lays the groundwork for recognising the emergence of lineage plasticity in lung cancer, especially when cancer cells may undergo similar developmental stages and lineage transformations during tumour formation. This increasing knowledge of lung lineage dynamics provides critical insights into the mechanisms underlying the adaptability of lung epithelial cells, which are capable of undergoing diverse differentiation processes.

The epithelial tissue of the lung is composed of a variety of cell types, intricately interconnected through a complex network of cellular differentiation. Within this network, certain highly stem‐like and self‐renewing cells, such as BASCs, basal cells, club cells and PNECs, possess the ability to differentiate into various terminally differentiated epithelial cells under both physiological and pathological conditions.^26^, ^28^ For instance, basal cells can differentiate into ciliated cells, NE cells and club cells, depending on the signalling cues in their microenvironment, playing critical roles in lung tissue repair and maintenance.^50^ Particularly in the context of lung injury, PNECs have the potential to differentiate into club cells and ciliated cells to contribute to the repair of damaged epithelial tissue.^51^ This differentiation network not only reveals the dynamic conversion capabilities of lung epithelial cells but also provides important insights into potential tumour‐originating cells.

In addition to the well‐characterised AT2 cells, BASCs, club cells, goblet cells and ciliated cells have also been implicated as potential cell‐of‐origin for LUAD.^52^, ^53^ For LUSC, candidate tumour‐initiating cells include basal cells, club cells and AT2 cells, which may undergo a transformation from normal differentiated cells to tumourigenic cells during cancer development.^50^, ^54^, ^55^ The discussion above on lung development and cell origin indicates that the onset of lung cancer largely follows the logical framework set by developmental lineages. The characteristics and behaviours of tumour subtypes are closely related to their cell origin and developmental trajectory: any bias or imbalance in lineage differentiation during normal development can potentially lead to tumour formation in the corresponding lineage. This phenomenon suggests that tumourigenesis in lung cancer is clearly constrained by lineage (Figure 1C). The differentiation of these cell types is not confined to the initiation of tumours but continues throughout the histological progression and transformation of the tumour. A comprehensive understanding of these classic cellular differentiation networks not only provides the molecular foundation for understanding tumour initiation but also offers important insights into tumour evolution and transformation. By delving deeper into these differentiation mechanisms, we can better comprehend the lineage plasticity and the differentiation diversity of tumour cells, and how these factors influence tumour therapy outcomes.

### 2.2 Characteristic changes induced by lineage plasticity in lung cancer

LUAD, LUSC and SCLC are the three primary types of lung cancer. Research has demonstrated that lineage plasticity in lung cancer permits tumour cells to transition from one histological type to another under specific conditions through alterations in TFs and signalling pathways.^56^, ^57^ For instance, the activation of SOX2 promotes the transformation of LUAD into squamous cell carcinoma, whereas the inactivation of *TP53* and *RB1* is crucial for the conversion of LUAD into SCLC.^58^, ^59^ In rare cases, there have even been reports of SCLC transforming into squamous carcinoma.^60^ These transformations enable tumour cells to shift from their primitive lineage characteristics to a state of high plasticity, allowing them to adapt to therapeutic interventions and survival pressures.

This direct and extensive transition between histological types is commonly attributed to tumour lineage plasticity. However, such plasticity is not confined to this. During the early stages of lung cancer formation and tumour progression, tumour cell lineages are continually modified to varying extents. It has been demonstrated that in the presence of specific driver mutations, club cells in the mouse lung can develop into squamous cell carcinoma upon SOX2 up‐regulation (in combination with *LKB1* deletion) and into LUAD upon NKX2‐1 regulation (in combination with *EGFR* mutation), each developing a distinct immune microenvironment corresponding to these histological types of lung cancer.^58^, ^61^

Club cells lose their original lineage identity through epigenetic transformation and acquire an AT2‐like phenotype upon oncogenic transformation.^13^ In vivo studies of LUAD have shown dysregulated growth signalling and lineage identity following P53 inactivation due to an aberrant AT1 differentiation program.^62^ These findings suggest that tumours are regulated in a lineage‐specific manner during early formation. Tumour cells initiate aberrant differentiation programs, resulting in the amplification or reduction of certain cellular lineage features and the formation of atypical characteristics.

To further elucidate the specific pathways of lineage plasticity in tumourigenesis, current studies have coined the term ‘lineage imbalance’ to describe this aberrant differentiation based on lineage traits.^5^, ^63^ This phenomenon occurs frequently and continuously throughout tumourigenesis and progression, gradually enhancing phenotypic diversity, a process recognised as lineage plasticity. Notably, the initial stages of this process, including the transformation of normally differentiated cells into intermediate cells and the arrested differentiation of these intermediate cells, are also observed in benign lesions such as pneumonia and acute lung injury.^64^, ^65^

Evidence suggests that the initial lineage characteristics of early tumours and precancerous lesions, once established, usually do not persist as the tumour progresses.^66^, ^67^, ^68^ There is a marked difference in the imaging features between early‐stage and advanced LUAD. As histological progression ensues, LUADs consistently exhibit invasive features such as solidity, cavitation and spiculation. Histopathologically, the structure of early lesions gradually degenerates, transitioning from a lepidic type to acinar and micropapillary forms, eventually evolving into a solid type.^63^, ^69^, ^70^ Throughout this process, early lineage factors and features progressively vanish.^63^, ^69^ Some highly invasive tumours even escape the regulation of their original lineage at an early stage.^71^, ^72^

## 3 INTRINSIC AND EXTRINSIC FACTORS INDUCING LINEAGE PLASTICITY IN LUNG CANCER

### 3.1 Genomic characterisation and epigenetic regulation

#### 3.1.1 | Epigenetic regulators of lineage plasticity

**FIGURE 2:**
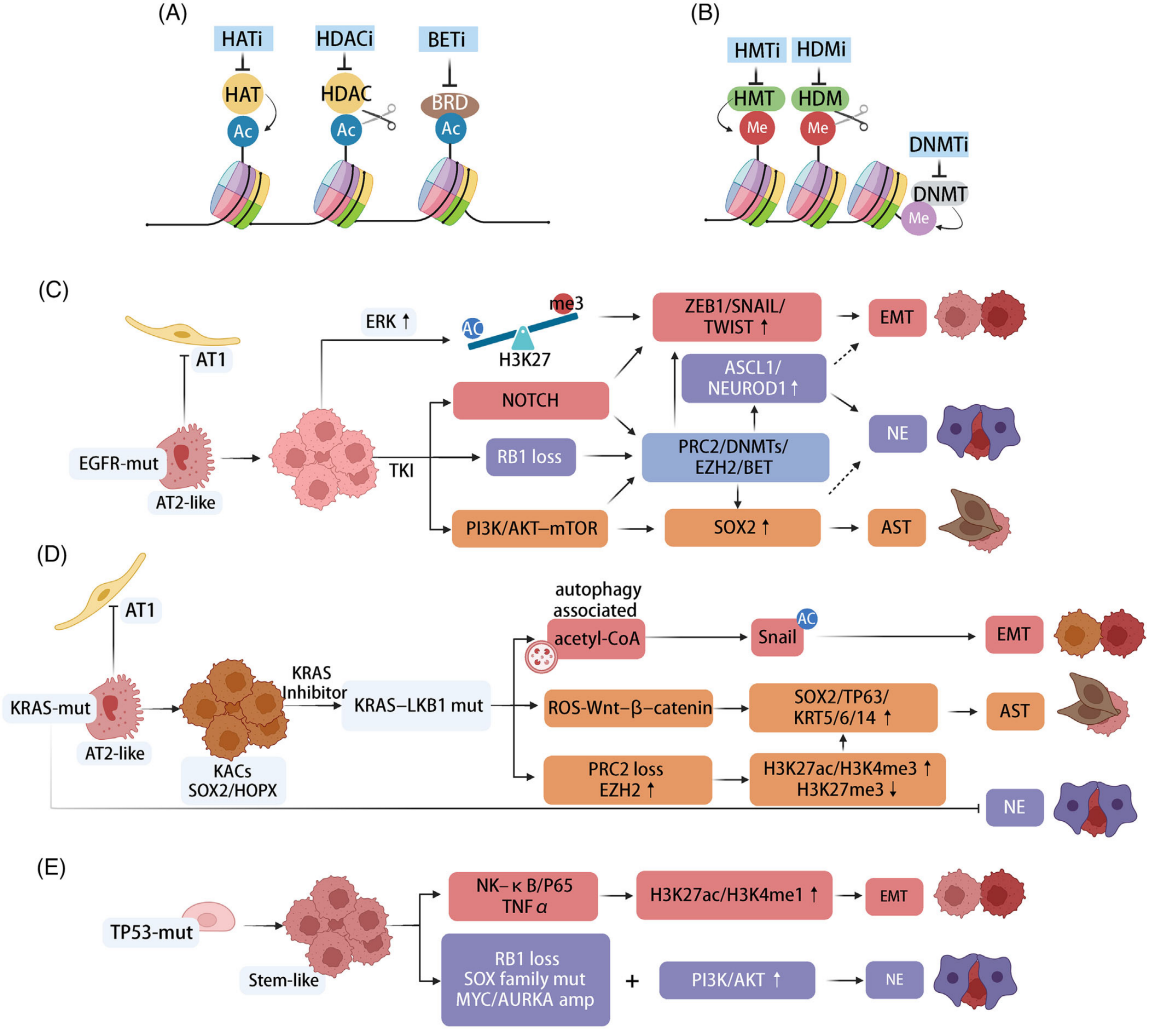
The impact of key gene mutations and epigenetic regulators on tumour lineage specification during tumourigenesis and progression. (A and B) Epigenetic modifiers and potential targeted drugs. (C) Lung‐originating precancerous cells carrying *EGFR* mutations tend to differentiate into AT2‐like cells, while inhibiting differentiation into AT1 cells. Tumour cells after progression or resistance enhance lineage plasticity through pathways such as ERK, NOTCH, PI3K/AKT–mTOR and *RB1* loss, and further promote lineage transformation via epigenetic pathways mediated by PRC2/DNMTs/EZH2/BET. (D) Lung‐originating precancerous cells with *KRAS* mutations form a unique SOX2^+^HOPX^+^ subpopulation, which is prone to acquiring *LKB1* mutations after progression and undergo mesenchymal or squamous transformation through autophagy, ROS–Wnt–β‐catenin pathways. (E) *TP53* mutations are associated with stem‐like characteristics in tumour cells and drive mesenchymal and NE transformation through inflammatory pathways such as NK‐κB/P65, TNFα up‐regulation and acquired genomic alterations (*RB1* loss, *SOX* family mutations, *MYC/AURKA* amplification, etc.). *Abbreviations*: HAT: histone acetyltransferase, HDAC: histone deacetylase, HATi: histone acetyltransferase inhibitors, HDACi: histone deacetylase inhibitors, BETi: bromodomain and extra‐terminal domain proteins inhibitors, HMT: histone methyltransferase, HDM: histone demethylase, DNMT: DNA methyltransferase, HMTi: histone methyltransferase inhibitors, HDMi: histone demethylase inhibitors, DNMTi: DNA methyltransferase inhibitors. Brick red represents EMT‐related pathways; purple represents NE‐related pathways; orange represents AST‐related pathways; grey‐blue represents epigenetic pathways associated with multiple transformations. Created in https://BioRender.com.

The transformation of non‐SCLC (NSCLC) entails intricate epigenetic reprogramming. During this cellular transformation, epigenetic modifications, including DNA methylation, histone modifications and chromatin remodelling, play pivotal roles in regulating gene expression and determining cell fate by establishing a dynamic yet stable regulatory environment^58^ (Figure 2A,B).

DNA methylation, a vital epigenetic factor regulating tumour profiles. Aberrant hypermethylation of promoter CpG islands frequently silences crucial tumour suppressors and lineage‐defining TFs. For example, hypermethylation of NKX2‐1 (TTF‐1) is associated with diminished alveolar lineage identity, promoting transdifferentiation towards a squamous phenotype.^58^ Similarly, hypermethylation of MGMT and MLH1 promoters impairs DNA repair mechanisms, promotes genomic instability and synergises with lineage dysregulation.^73^ This process is primarily mediated by DNA methyltransferases (DNMT1, DNMT3A and DNMT3B), which lock cells into progenitor‐like or alternative phenotypic states and facilitate lineage transdifferentiation, with DNMT1 identified as a major mediator of plasticity.^74^ SCLC is characterised by elevated DNMT expression and a DNA methylation profile similar to that of NEPC, including hypomethylation of neuronal TFs ASCL1, HES6 and ONECUT2.^75^, ^76^ In NSCLC, global hypomethylation of genomic regions encoding lineage plasticity drivers, such as ASCL1 and NEUROD1 in the NE program, promotes their aberrant expression and facilitates transformation to SCLC.^77^ In SCLC, defects in KMT2C lead to histone and DNA hypomethylation through DNMT3A‐mediated epigenetic reprogramming, further promoting cancer metastasis.^78^ DNMT3A also drives stemness expression in NSCLC by up‐regulating Wnt/β‐catenin signalling.^79^ The DNMT3A inhibitor miR‐708‐5p has demonstrated substantial reductions in stemness characteristics.^80^ Such environment‐dependent methylation redistribution marks lineage pathways, driving phenotypic plasticity and heterogeneity.

Similar to lineage‐defining factors like NKX2‐1 and SOX2, tumour lineage is governed by binary epigenetic regulation. Post‐translational modifications that promote transcriptional activity (e.g., histone H3 lysine 4 aminomethylation [H3K4me1] or trimethylation [H3K4me3]) and those that repress transcription (e.g., H3K27me3) are concurrently enriched, forming binary chromatin.^81^, ^82^ Understanding the intricate mechanisms governing cellular plasticity necessitates a deep dive into the modifications of histones, collectively known as the ‘histone code’. A key feature within this code is the concurrent presence of transcriptionally active marks, like H3K4me1 or H3K4me3, and repressive marks, such as H3K27me3. This state, known as bivalent chromatin, is pivotal in regulating the expression of genes linked to plasticity. Typically, genes with bivalent marks are maintained at low transcription levels, which can be up‐regulated by the loss of H3K27me3 or down‐regulated by the loss of H3K4me3, a condition termed as being transcriptionally ‘equilibrium’.^83^ During normal development, bivalent chromatin is predominantly found at regulatory regions of lineage‐specific TFs and other developmental genes, facilitating swift and flexible control over cellular states.^84^

EZH2, a crucial enzyme of the polycomb repressive complex 2 (PRC2), facilitates the addition of H3K27me3 marks. Overexpression of EZH2 deposits repressive H3K27me3 marks on differentiation‐associated genes, maintaining stem‐like plasticity and biasing cells towards squamous or NE fates.^85^ Suppressing EZH2 function has been observed to obstruct and, at times, revert epithelial–NE transformation in human and murine models of prostate carcinoma and SCLC.^86^, ^87^ LSD1, another significant player in chromatin dynamics, is a demethylase acting on H3K4me1, H3K4me2, H3K9me1 and H3K9me2.^88^ In embryonic stem cells, LSD1 actively removes activating histone marks (H3K4me1/2) from key developmental loci, thereby unlocking lineage transitions via lineage‐specific TFs like FOXA2, orchestrating pluripotency and cellular potential.^89^ In *EGFR*‐mutant NSCLC, LSD1 suppression restores H3K4 methylation at alveolar gene promoters, reversing epithelial–mesenchymal transition (EMT) and resisting NE plasticity.^90^, ^91^ Considering LSD1's pivotal function in sustaining pluripotency, it is predictable that its dysregulation in malignancies is associated with EMT), epithelial–NE adaptability and the sustenance of cancer stem cell (CSC) traits. Additionally, dynamic regulation by KDM5A, histone acetyltransferases (e.g., p300/CBP) and histone deacetylases (HDACs) influencing chromatin accessibility at lineage enhancers should not be overlooked. In LUAD, decreased HDAC2 activity, often due to smoking, causes hyperacetylation at inflammation and EMT gene promoters, promoting mesenchymal plasticity and immune evasion.^92^ Conversely, inhibition of *EGFR* or *KRAS* enriches H3K27ac at NE lineage enhancers (e.g., ASCL1), initiating SCLC transformation.^93^, ^94^ Thus, targeting HDACs or p300 holds potential for modulating plasticity trajectories and restoring lineage fidelity.

Finally, mutations or loss of chromatin remodellers like SWI/SNF complex subunits (*SMARCA4*, *ARID1A*) are crucial in epigenetic plasticity. In LUAD and LUSC, loss of *SMARCA4* alters nucleosome positioning and enhancer identity, reducing accessibility of alveolar differentiation enhancers and activating squamous TF networks (e.g., TP63, SOX2), accelerating histological transition and hindering epithelial repair. Additionally, molecules that regulate chromatin structure and accessibility, including LKB1, KMT2D, SETDB1 and so on, have been reported to be able to influence the epithelial expression program of lung cancer and determine its differentiation fate.^95^, ^96^, ^97^, ^98^

#### 3.1.2 | Lineage plasticity and genomic features

In the context of epigenetic initiation, different histological types of lung cancer each exhibit distinct genomic characteristics, such as *RB1* and *MYC* alterations in SCLC, NFE2L2, TP63 and NOTCH1 variations in LUSC, and *EGFR*, *KRAS* and *ERBB2* variations in LUAD. High‐frequency driver variations such as *TP53*, *CDKN2A*, *PIK3CA* and *PTEN* are common across these histological types.^99^, ^100^, ^101^ Post‐transformation samples typically retain the driver mutations of their original histological type, though exceptions exist.^102^ For instance, some NSCLC and SCLC samples share other alterations without detectable driver mutations, suggesting the possibility of driver mutations being lost during transformation.^103^ Nonetheless, the majority of cases retain the same primary driver mutation before and after transformation. The lack of concordance in major mutations in all transformed cases may relate to the two hypothesised pathways of transformation. Additionally, mutations in primary oncogenic drivers can influence the direction of transformation to various histological types.^6^

*EGFR* encodes a receptor tyrosine kinase that activates RAS–RAF–MEK–ERK and PI3K–AKT–mTOR signalling cascades through ligand‐induced dimerisation and autophosphorylation, crucial for lung epithelial cell proliferation and differentiation. EGF signalling selectively regulates AT2 proliferation, and activating *EGFR* mutations (such as exon *19 deletion* and *L858R*) maintain receptor constitutive activation, sustaining epithelial phenotype while blocking alveolar differentiation (AT2→AT1).^6^, ^104^ This partially explains why early‐stage *EGFR*‐mutated LUAD exhibits higher differentiation and better prognosis. This unique LUAD subgroup is notable for lineage transformation during progression and drug treatment. EMT is clearly evident during *EGFR*‐mutated LUAD tumourigenesis, as studies by de Miguel et al.^105^ and Inoue et al.^94^ demonstrated following *EGFR* mutation or ERK activation, chromatin remodelling elevates H3K27ac at enhancers of progenitor‐/stemness‐related and EMT genes (e.g., ZEB1, SNAI2) while dynamically modulating the H3K27ac/H3K27me3 balance, thereby sustaining a permissive epigenetic landscape that promotes lineage plasticity. Reduced H3K27me3 at NE genes also occurs during *EGFR*‐mutant progression or transformation.^106^ These findings indicate that *EGFR*‐mutant tumour cells utilise histone modification‐related epigenetic pathways for lineage shifts, more pronounced under drug therapy.^107^ Studies by Alvaro et al.^108^ observed enhanced expression of PRC2 complex, PI3K/AKT and NOTCH pathway genes, and PI3K/AKT pharmacological inhibition delayed tumour growth and NE transformation in *EGFR*‐mutant patient‐derived xenografts. Hu et al.^109^ confirmed the presence of ASCL1/NEUROD1 enhancer subpopulations in *EGFR*‐mutant cells, mediating drug resistance and EMT via high ASCL1 expression.^110^ Additionally, *EGFR*‐TKI treatment triggers rapid chromatin remodelling via DNMTs, EZH2 and BET proteins and achieves the acquisition of drug resistance through identity transformation.^111^, ^112^, ^113^, ^114^ Reports also suggest that *EGFR*‐mutant cells resist targeted therapy via SWI/SNF chromatin remodelling complexes through mutations in chromatin structure‐related factors like SMARCA4/SMARCA2^105^ (Figure 2C).

Similarly, *KRAS* mutations significantly impact lineage plasticity via signal and epigenetic landscape alterations. *KRAS* encodes a GTPase mediating critical signalling pathways such as MAPK/ERK and PI3K/AKT, essential for cell proliferation, differentiation and survival. *KRAS* mutations, especially at codon *G12* (*G12C*, *G12V*, *G12D*), disrupt alveolar epithelial differentiation and selectively induce alveolar epithelial cells towards AT2 lineage, generating progenitor‐like KRT8‐positive alveolar cells (KACs) with increased plasticity markers (SOX2, HOPX).^27^, ^104^ During tumourigenesis, *KRAS*‐mutant NSCLC activates ATF4‐mediated integrated stress responses under nutrient stress, sustaining tumour proliferation and progression via KRAS–NRF2–ATF4 signalling.^115^ Under *KRAS* inhibitor therapy, lineage plasticity programs activated by *KRAS–LKB1* co‐mutation shift metabolism and oxidative stress signals, promoting squamous lineage via ROS–Wnt axis‐driven adeno‐to‐squamous transition (AST).^116^, ^117^, ^118^ *KRAS–LKB1* co‐mutant cells also use autophagy‐derived acetyl‐CoA to acetylate and stabilise EMT TF Snail.^119^ These studies highlight unique plasticity pathways in *KRAS*‐mutant tumours. However, LUADs that transform into SCLC exhibit fewer *KRAS* mutations, indicating that *KRAS*‐mutant LUADs are less likely to undergo NE transformation.^108^ This also indicates that some mutations act as limiting factors in the transformation process (Figure 2D).

Finally, *TP53* mutations consolidate lineage plasticity, promoting aggressive tumour behaviour and therapeutic resistance. *TP53* encodes a crucial tumour suppressor protein that regulates genomic stability, apoptosis and cell differentiation.^62^, ^120^ *TP53* mutations severely disrupt lineage fidelity, unleashing aberrant differentiation programs. Mutant p53 promotes squamous lineage transformation by suppressing alveolar fate regulators (NKX2‐1) and activating squamous cell enhancers (TP63, SOX2), thereby rewiring transcriptional networks towards stemness and plasticity.^58^, ^68^, ^121^ Additionally, mutant p53 facilitates lineage plasticity through epigenetic mechanisms, including EZH2‐driven deposition of repressive H3K27me3 marks and LSD1‐mediated removal of activating marks (H3K4me1/2), stabilising a mesenchymal state.^77^, ^122^, ^123^ While *TP53* mutations drive adenocarcinoma‐to‐squamous lineage transition, these mutations also cooperate with various genetic and signalling pathway alterations, collectively promoting transformation to SCLC. Particularly, loss of *TP53* and *RB1*, mutations in SOX family genes, activation of the PI3K/AKT pathway and amplification of *MYC* and *AURKA* collectively drive SCLC features^77^, ^124^ (Figure 2E). Especially in *EGFR*‐mutant tumours, *RB1* inactivation via complex rearrangements appears to favour SCLC/LUSC transformation under growth inhibitory pressure,^125^ but this transformation has no significant association with *EGFR* mutations and is often accompanied by the down‐regulation of EGFR expression.^6^, ^108^, ^126^ Thus, *RB1* inactivation is predictive of the risk of LUAD transforming into more aggressive lung cancer types.^125^ A retrospective study revealed that *RB1* and *TP53* were the most prevalent mutations in patients with *EGFR*‐mutant NSCLC converting to SCLC, accounting for 68% and 36% of cases, respectively.^127^ High‐frequency mutations emerging post‐transformation include PTEN.^128^

During the progression of histological subtypes of LUAD (lepidic, papillary, acinar, micropapillary, solid), high‐grade tumour cells exhibit a high degree of chromosomal complexity, with a greater burden of loss of heterozygosity and subclonal somatic cell copy number alterations.^129^ The low clonal diversity in high‐grade regions implies substantial proliferation of cells in a short period. Higher frequencies of truncal arm or focal 3q gains, as well as *SMARCA4* gene alterations, were observed in less differentiated solid‐type regions.^130^ Dysfunction in these specific chromosomal regions and driver genes associated with cell differentiation fate is typically accompanied by highly aggressive behaviour and further promotes metastasis. However, further mechanism research is needed to fully understand these key gene mutations’ specific contributions in lineage transformation. As tumour cells adapt to the metastatic tissue environment, the genome of metastases retains some similarity to the primary tumour, typically exhibiting a higher variant allele frequency and tumour mutation burden. Whether genome doubling generates metastatic potential or merely reflects elevated chromosomal instability remains unknown. This increased instability may accelerate cancer genome evolution, allowing tumours to explore greater evolutionary landscapes. A recurring theme in cancer involves the disruption of chromatin modifications in the late stages of the disease, which may contribute to relaxing constraints on cancer genomes.

Overall, the role of driver gene mutations is lineage dependent – identical genetic alterations may trigger distinctly different tumour phenotypes depending on the cellular lineage context. At the same time, lineage transformation does not occur randomly but follows specific trajectories and mechanisms: for example, the lineage transformation of LUAD to SCLC or LUSC follows clear molecular pathways, rather than being a mere random event. The lineage dependence of driver mutations and the non‐random nature of lineage transformation further highlight the intrinsic procedures behind the plasticity of lung cancer lineages.

### 3.2 Microenvironmental factors and mechanisms associated with lineage plasticity

Tumour‐associated macrophages (TAMs) play a crucial role in EMT in solid tumours such as NSCLC through various mechanisms. The enrichment of TAMs and the enhanced expression of their markers, such as CD68 and CD163, are closely related to the down‐regulation of E‐cadherin, and the up‐regulation of vimentin and matrix metalloproteinases (MMPs), all of which are characteristic of the EMT phenotype.^131^ TAMs secrete a variety of pro‐inflammatory and growth factors, including TGF‐β, IL‐6, CCL2, IL‐10 and MFG‐E8, which activate multiple EMT‐associated signalling pathways, including TGF‐β/SMAD/ZEB, COX‐2/PGE2, ATM/NF‐κB, JAK1/STAT3, CRYAB/ERK1/2/Fra‐1/Slug and β‐catenin pathways.^132^, ^133^, ^134^ These pathways work together to suppress epithelial markers and promote mesenchymal marker expression, thereby inducing the loss of cell polarity and acquisition of the mesenchymal phenotype, enhancing tumour cell migration, invasion and metastatic ability. Notably, TGF‐β plays a dual role: it is integral to erlotinib resistance, EMT and IL‐6 axis activation in drug‐resistant bronchoalveolar metastatic cancer H1650 cells.^135^, ^136^ Silencing TGF‐β1 reverses EMT, thereby enhancing the sensitivity of A549/DDP cells to cisplatin.^137^ In advanced tumours, TGF‐β transforms into a pro‐cancer factor through pathways such as AKT and C‐jun/SMAD3, further promoting angiogenesis, immune evasion and activation of Cancer‐Associated Fibroblasts (CAFs).

**FIGURE 3:**
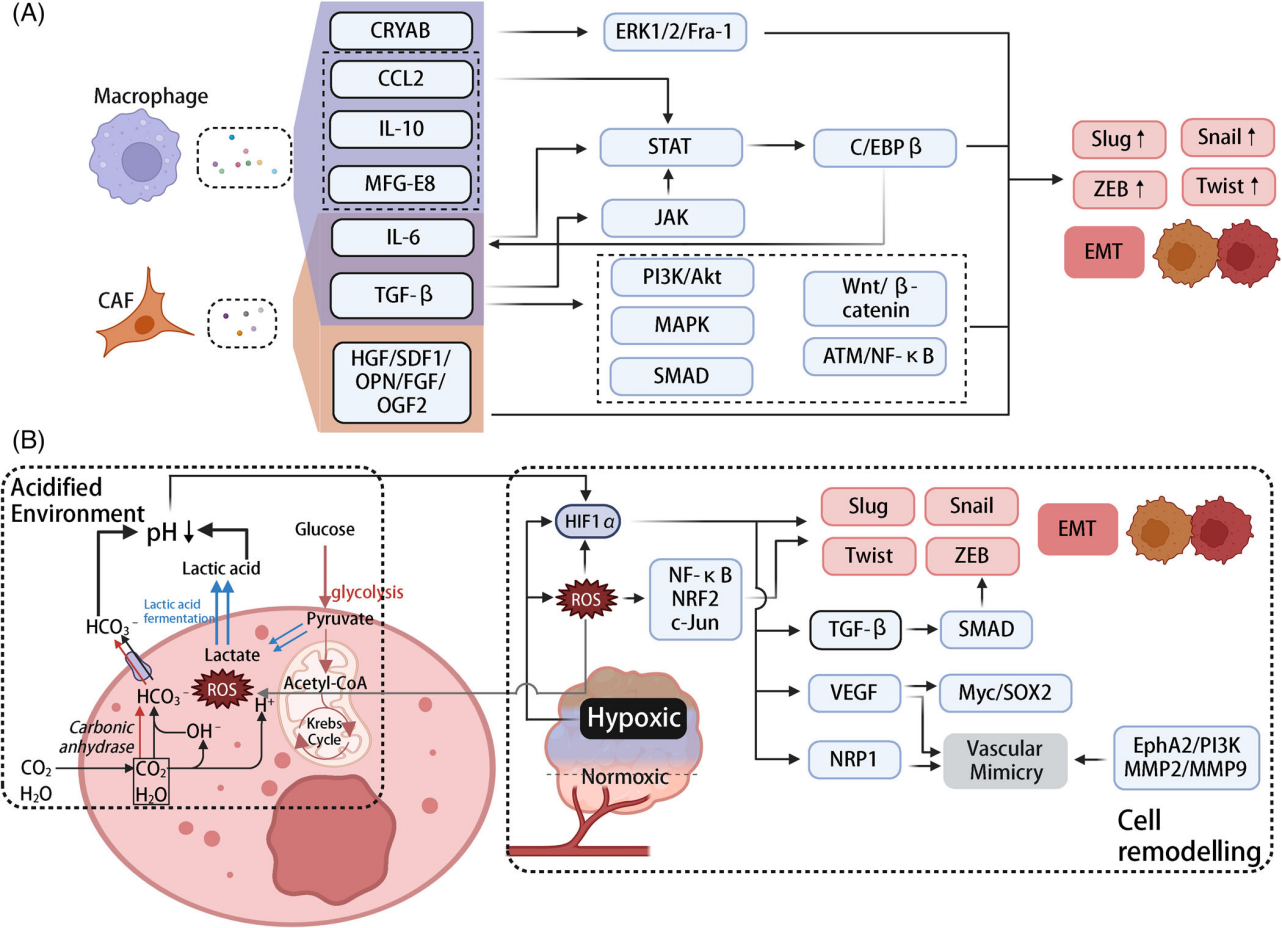
Tumour epigenetic regulation and tumour microenvironment. (A) The soluble cytokines (TGF‐β/IGF2/HGF/SDF1/OPN/FGF/IL‐6/MFG‐E8, etc.) from TAMs and CAFs in the microenvironment affect signalling pathways such as MAPK, PI3K/Akt and Wnt/β‐catenin, mediating EMT. (B) ROS produced in the hypoxic environment and up‐regulated HIF1α promote tumour cell morphological and lineage transformation. Additionally, the decrease in cell environment pH caused by extracellular lactate accumulation induced by ROS further stabilises HIF1α expression. Created in https://BioRender.com.

Additionally, TAMs form multiple positive feedback loops with lung cancer cells, accelerating the progression of EMT. Mechanistically, IL‐6 secreted by TAMs can promote β‐catenin nuclear translocation through the COX‐2/PGE2 pathway, while the IL‐6/STAT3‐C/EBPβ loop further sustains its high expression.^138^, ^139^ CCL2 down‐regulates E‐cadherin and up‐regulates MMP‐2/MMP‐9, vimentin and Twist expression, while synergising with IL‐6 to enhance STAT3 phosphorylation, thereby driving the EMT cascade.^140^ IL‐10 also activates the STAT3 pathway and participates in EMT.^141^ Furthermore, TAMs secrete MFG‐E8, which activates the Stat3 and Sonic Hedgehog pathways, providing survival signals to CSCs and enhancing drug resistance.^142^ These mechanisms form the critical network through which TAMs promote EMT and CSC transformation, highlighting their multi‐faceted role in lung cancer progression (Figure 3A).

The influence of microenvironmental components on tumour cells extends beyond the induction of lineage imbalances, encompassing the selection of confounding phenotypes. Recent evidence indicates that in mixed‐state tumours with numerous lineage clones, NK cells differentially target tumour cells at various developmental stages based on the expression of tumour TFs such as SOX2 and SOX9.^143^ EMT is also associated with an increased expression of various immune‐suppressive cytokines, such as the overexpression of immune checkpoint molecules like CTLA‐4 and TIM‐3, which are related to EMT in NSCLC. There may be an association between immune rejection and EMT in NSCLC. Further studies are needed, as the underlying mechanisms are not yet fully understood. A deeper understanding of this association could drive the development of biomarkers capable of accurately predicting the efficacy of immunotherapy.^144^

The most extensively studied cytokine secreted by CAFs is TGF‐β, which regulates other cancer‐associated pathways such as MAPK and PI3K/Akt through non‐canonical signalling mechanisms.^145^ These pathways, when activated in CAFs, also influence the state of tumour cells. Zhang et al.^146^ used CUDC‐907 to target the PI3K/AKT pathway in CAFs and successfully inhibited cancer progression. These pathways are modulated by growth factors and inflammatory mediators commonly secreted by CAFs, including hepatocyte growth factor (HGF), stromal‐derived factor‐1 alpha, osteopontin (OPN), fibroblast growth factor (FGF) and IL‐6^133^, ^147^, ^148^, ^149^, ^150^ (Figure 3A).

The most extensively studied cytokine secreted by CAFs is TGF‐β, which regulates other cancer‐associated pathways such as MAPK and PI3K/Akt through non‐canonical signalling mechanisms.^145^ The effects driven by TGF‐β are highly cell type dependent. Despite the dual roles of the TGF‐β pathway at different stages of tumour progression, its unregulated presence in many cancers makes it a focal point in oncology. In healthy tissues and early tumour formation, TGF‐β activation induces protective effects such as cell cycle arrest and apoptosis.^151^ Conversely, in advanced cancers, TGF‐β induces reprogramming of intracellular amino acid metabolism, promoting the acquisition of a mesenchymal phenotype in NSCLC cells, thus driving metastatic disease.^152^ Additionally, IL‐6 plays an important role. Shintani et al.^150^ have confirmed that IL‐6 secreted from CAFs mediates chemoresistance in NSCLC by inducing EMT. Furthermore, classic studies have found that in NSCLC, insulin‐like growth factor 2 (IGF2) released by CAFs can induce stem cell transcription programs, driving EMT.^153^ In other cancers, such as liver cancer, studies have shown that IGF2 promotes plasticity progression through the TF NANOG.^154^ NANOG expression is linked with the development and maintenance of a pluripotent state in CSCs and acts as a co‐activator of hypoxia‐inducible factor 1 (HIF‐1) transcription by telomerase reverse transcriptase, playing a crucial role in telomerase activity for telomere extension.^155^, ^156^, ^157^

Soluble pro‐inflammatory factors are typically produced at high levels in cancer cells but can also originate from other tumour microenvironment components, most notably myeloid cells and CAFs. In addition to the soluble pro‐inflammatory factors secreted by myeloid cells and CAFs, the stromal properties of the tumour microenvironment modulate transcription and epigenetic plasticity in cancer cells. A stiff tissue matrix not only increases the risk of carcinogenesis but also promotes cytokine production, such as IL‐23, by activating the focal adhesion kinase (FAK) and extracellular signal‐regulated kinase (ERK) signalling pathways.^158^, ^159^, ^160^ In breast cancer, the Benedetti laboratory and others have shown that integrins in cancer cells sense tumour environment stiffness and mediate the activation of key plasticity TFs such as Twist and YAP/TAZ, leading to the EMT transcriptional programme.^161^, ^162^

Notably, hypoxia is intricately linked to the epigenetic and transcriptional reprogramming of cancer cell plasticity. Under hypoxic conditions, HIF‐1α is stabilised and translocated to the nucleus, where it activates the expression of core EMT TFs such as Twist, ZEB1, Snail/Slug and lysyl oxidase by binding to hypoxia response elements. These proteins work together to promote cytoskeletal remodelling and EMT.^163^, ^164^, ^165^ HIF‐1α also induces TGF‐β, which in turn enhances the EMT signalling network through the SMAD–ZEB axis. Additionally, HIF‐1α can cross‐talk with Notch signalling, enhancing EMT transcriptional activity via the Notch–RBPJ complex.^166^

At the microenvironment level, hypoxia remodels the ECM and vascular structures. HIF‐1α enhances the secretion of VEGF by stromal cells (such as CAFs and endothelial cells), which not only promotes angiogenesis but also activates Myc and SOX2 signalling within tumour cells. This initiates transcriptional programs associated with the CSC phenotype, reinforcing stemness and plasticity characteristics.^167^ More importantly, under hypoxic conditions, HIF‐1α can enhance the vasculogenic mimicry (VM) ability of LUAD cells by up‐regulating NRP1 (Neuropilin‐1), a process that involves VEGF‐regulated vascular‐like structure formation, aiding perfusion and invasive spread in hypoxic regions.^168^ At the same time, hypoxia drives the expression of VEGF and VE‐cadherin in VM, with pathways such as EphA2/PI3K and MMP‐2/MMP‐9 coordinating ECM remodelling and channel formation, allowing cancer cells to acquire endothelial‐like traits and form vascular‐like structures, further supporting metastasis and resistance^169^, ^170^, ^171^ (Figure 3B).

Hypoxia also increases ROS levels in cancer cells. ROS, by inhibiting PHD enzymes, activating HIF‐1α and triggering the co‐activation of redox‐sensitive TFs (including NF‐κB, Nrf2 and c‐Jun), promote EMT, CSC phenotype and metabolic reprogramming (such as enhanced glycolysis and inhibited mitochondrial respiration).^172^ Nrf2 acts as an upstream regulator of HIF‐1α transcription, activating HIF1A expression through ARE binding sites, while stabilising HIF‐1α signalling by up‐regulating ROS scavenging systems (such as TXNRD1, NQO1 and HO‐1) and regulating glycolysis and the pentose phosphate pathway (PPP). High NADPH supply enhances antioxidant capacity and CSC maturity.^173^ During this process, hypoxia also enhances the transcriptional activity of SOX9 by promoting its lactylation modification, further sustaining the activation of glycolytic programs and strengthening stemness and EMT phenotypes via SOX9‐mediated transcriptional networks, forming a positive coupling between lactate metabolism and stemness phenotype.^174^

Additionally, a decrease in pH is another important feature of the hypoxic microenvironment. Under low pH conditions, CA IX/XII and lactate dehydrogenase catalyse lactate production and extrusion, inducing environmental acidification (pH ∼6.5 ± 0.3). This process is accompanied by ECM remodelling, MMP activation and immune function suppression, reducing the cytotoxicity of CD8⁺ T cells, promoting immune escape and enhancing tumour cell resistance.^175^, ^176^, ^177^, ^178^, ^179^, ^180^, ^181^ Acidification can also further stabilise HIF‐1α, creating a positive feedback loop (Figure 3B).

These mechanisms collectively form a multi‐layered network: (1) transcriptional/epigenetic reprogramming – HIF‐1α → EMT/CSC; (2) matrix remodelling and VM – VEGF/VE‐cadherin/MMP; (3) metabolic reprogramming – ROS/Nrf2/HIF‐1α/glycolysis/PPP; (4) immune suppression – pH reduction and T cell dysfunction. This integrated regulation collectively drives EMT, CSC evolution and histological transition under hypoxic conditions, significantly enhancing proliferative and invasive abilities while also exacerbating therapeutic resistance.

## 4 LINEAGE PLASTICITY PERSISTS DURING THE ORIGIN AND DEVELOPMENT OF LUNG CANCER CELLS

**FIGURE 4:**
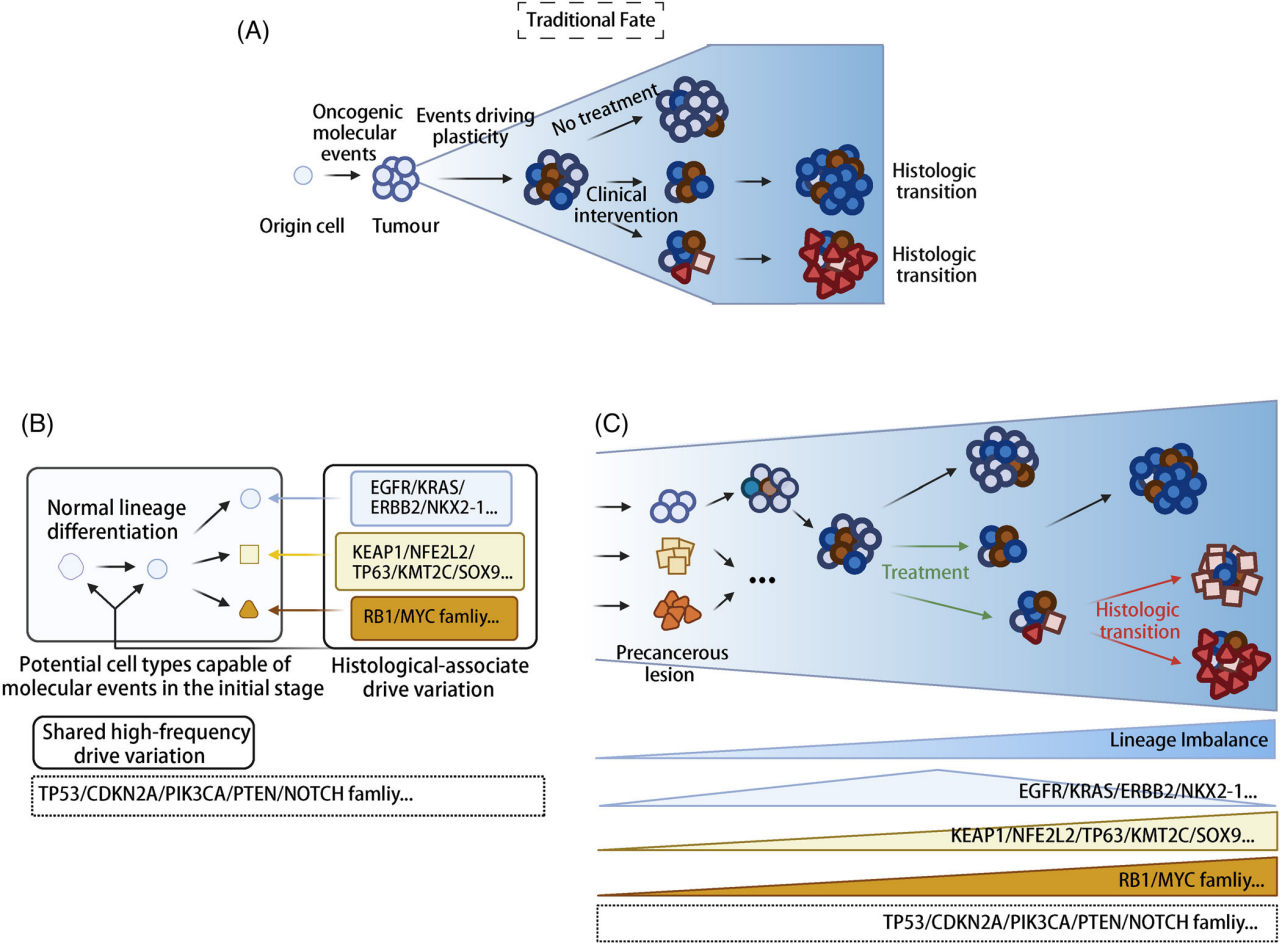
Tumour cell lineage fate landscape. (A) Traditional histological fate diagram of tumour cells. (B) Specific types of lung epithelium with corresponding driver mutations mediate tumour formation. Undifferentiated cells that acquire specific driver mutations lead to differentiation into particular lineages. (C) The updated tumour cell evolution diagram shows that the lineage imbalance of the cell types capable of tumour formation continues to occur after carcinogenic transformation, and the Histologic transition is finally realised along with clinical intervention and multi‐node genomic variation. The blue background indicates the strength of the lineage imbalance, the circle indicates alveolar cells, the square indicates squamous cells, the triangle indicates endocrine cells. Created in https://BioRender.com.

Studies on lung cancer have demonstrated that the classification of cancer DHS clusters is predicated on the functional attributes of the cancer rather than its developmental origins.^182^ Some tumour‐origin cells, even after undergoing driver mutations, still require specific lineage regulation to form nascent precancerous lesions or primary foci (Figure 4A,B). Maeda et al.^183^ pointed out that the respiratory epithelium in mouse with only oncogenic *Kras G12D* cannot induce mucinous adenocarcinoma; only when Nkx2‐1 expression is reduced (haploinsufficiency) and *Kras^G12D* is present can lesions resembling human mucinous adenocarcinoma form. This necessity is twofold: to adapt to the local physiological milieu and appropriately emulate the functions of normal cells and to exhibit molecular characteristics akin to normal cells at an early stage, thereby mitigating immune‐mediated damage. Furthermore, certain driver mutations achieve maximal oncogenic potential only when cells are lineage‐regulated to manifest a well‐defined cell type. This phenomenon has been experimentally confirmed in lung cancer research: Hill et al.’s^184^ study demonstrated that *EGFR* and *KRAS*‐driven mutations inherently exist in normal AT2 cells, but these cells do not develop into tumours unless they are reprogrammed into a progenitor‐like cell state. Only under specific microenvironmental stimuli (such as immune induction and lineage reprogramming triggered by PM2.5) are these cells activated and transformed into tumours^184^ (Figure 4).

While it is conceivable that tumour‐derived cells undergo driver mutations and form tumours directly without lineage differentiation regulation, empirical evidence from animal models and large‐scale population‐based cohorts suggests otherwise. The direct introduction of a driver mutation into a tumour‐derived cell is less effective in tumour formation compared with introducing a driver mutation into a subpopulation with potent progenitor cell functionality.^183^ Gardner et al.^57^ demonstrated in a mouse model that directly introducing *Myc*‐driven mutations into mature LUAD cells does not trigger histological transformation; only in subpopulations with strong progenitor‐like plasticity and basal lineage features can Myc tolerate and initiate the transformation from LUAD to SCLC. This implies that the commonly referenced tumour‐derived cells may not always be the true progenitors of the tumour. A classic study using a mouse airway model showed that even fully differentiated lung epithelial secretory cells can revert to a basal stem cell‐like state and regain progenitor functionality under basal stem cell damage or specific stimulus conditions.^185^ This suggests that the lineage state itself greatly influences the tumourigenic efficiency after driver mutations. Furthermore, Mainardi et al.^186^ performed lineage tracing in a *KRAS G12V*‐driven mouse LUAD model and found that the efficiency of tumour formation was significantly higher when mutations were introduced into club cells than when they were introduced into ciliated or Goblet cells, further demonstrating that the same mutation shows markedly different tumourigenic potential in different lineage backgrounds.

Mutant cells typically do not significantly deviate from normal differentiation pathways before tumour formation. Instead, they control their phenotype to an intermediate state between two normal cell types, effectively existing between normal and tumour states. This is akin to the intermediate alveolar cells observed in cancer‐free mice exposed to acute lung injury.^65^ Another recent study using a mouse AT2 cell‐based 3D organoid model demonstrated that the *Kras G12D* mutation can induce a ‘damage/plasticity’ intermediate state before tumour formation, maintaining cells in a state between AT2 and AT1 differentiation.^187^

Nonetheless, tumour cells harbouring different driver mutations exhibit varying degrees of differentiation from the outset of their formation. For instance, early‐stage *EGFR* mutant tumours display more complete epithelial characteristics compared with *KRAS* mutant tumours.^27^ In the pancreas *Kras G12D* mouse model, *EGFR* activation was similarly found to be a precursor to the formation of mPanIN (early epithelial metaplasia lesions).^188^ The plasticity pattern may be constrained by cell type, yet it is unequivocal that the mutated cells must possess some initial differentiation potential rather than being terminally differentiated^189^ (Figure 4B,C).

The aforementioned early lesions often exhibit high differentiation and are indicative of a relatively favourable prognosis. However, this initial lineage regulation is not perpetuated throughout tumour progression. As lineage regulation wanes, tumour cells increasingly display malignant phenotypes and enhanced adaptations to the microenvironment. The loss of initial features augments invasiveness and signifies a further remodelling of intrinsic cellular pliancy. This process engenders a polyclonal landscape where tumour cells with varying degrees of differentiation coexist across different differentiation pathways, thereby elevating the likelihood of metastasis and colonisation.

## 5 LINEAGE PLASTICITY UNDERLIES THE PROGRESSION OF LUNG CANCER

The dysregulation of tumour cell lineage stands as a pivotal factor in the development of lung cancer, manifesting through alterations in molecular characteristics and cell fate. Related studies have concentrated on the transformative processes of tumour cells, such as the conversion of adenocarcinoma to SCLC and adenocarcinoma to squamous carcinoma. The profound changes in cell morphology and molecular features observed during these transformations underscore the complexity and heterogeneity inherent in tumour progression.

### 5.1 Characteristic changes in cellular and molecular functions and histological phenotype transitions driven by lineage plasticity

SOX2 and NKX2‐1, as pivotal lineage‐defining TFs in lung epithelium, exert a decisive influence on the regulation of tumour cell fate.^58^, ^190^, ^191^ This transformation not only modifies the tumour immune microenvironment but also induces cellular phenotypic alterations, such as the morphological transition from adenocarcinoma to squamous carcinoma cells and the reorganisation of intracellular and intercellular signalling pathways.^192^ Mollaoglu's study supports this notion, proposing that in the transformation from adenocarcinoma to squamous carcinoma, the deletion of NKX2‐1 or overexpression of SOX2 recruits tumour‐associated neutrophils, with the CXCL5 chemokine further promoting SOX2‐driven squamous cell transformation.^58^ However, the simultaneous deletion of NKX2‐1 and overexpression of SOX2 are not both required to achieve this transformation. Indeed, the gain of TFs that enhance cellular plasticity and the loss of those that maintain the differentiated epithelial state converge to produce a distinct binary lineage switching. Nevertheless, this binary phenomenon may be rare, as further exploration revealed that the lineage fate of tumour cells is governed by the dynamic balance of lineage factors. Tang et al.^103^ demonstrated that upon imbalance of alveolar epithelial TFs (NKX2‐1 and FOXA2) and squamous TFs (TP63 and SOX2), cells finely regulate the lineage switch through neutrophil infiltration.

Notably, the phenotypic transition resulting from an imbalance of lineage factors is also evident in the transformation from adenocarcinoma to SCLC. Kong et al.^193^ demonstrated that the formation of SCLC‐Aα subtypes and the maintenance of their neuronal lineage status post‐transformation required the cooperation of core regulatory circuits involving NKX2‐1 and SOX1.^194^ ASCL1 also plays a crucial role in determining NE cell fate, being highly expressed in classical SCLC and LCNEC tumours that maintain NE characteristics.^195^ ASCL1 expression correlates with the tumour‐initiating capability of SCLC.^196^ Increasing evidence supports a tumour‐suppressive role for Notch‐1 signalling in NE tumours. Delta‐like protein 3 (DLL3), an atypical member of the Notch receptor ligand family, appears to inhibit Notch receptor activation, contrary to related family members. DLL3 is a downstream transcriptional target of ASCL1 that promotes NE tumourigenesis by inhibiting the Notch receptor pathway.^197^, ^198^

Other signalling pathways also play crucial roles in the transformation of adenocarcinoma to SCLC. For instance, up‐regulation of FGF9 significantly induces the expression of LUAD NE markers (e.g., ASCL1 and SYP), altering cell survival and proliferation characteristics, thus promoting tumour cell transformation.^199^ Longitudinal single‐cell transcriptome analyses of SCLC tumours revealed the role of MYC‐mediated activation of Notch signalling in facilitating the transformation of ASCL1‐positive SCLC to a non‐NE YAP1‐positive state.^200^ This subtype transition can be inhibited by Notch inhibitors. Both the PI3K/AKT and MYC pathways have been implicated in driving small‐cell transformation in *EGFR*‐mutant LUAD.^108^ Gardner et al.^57^ also demonstrated that successive shifts in cellular states can be observed in an animal model of targeted therapy. Activation of the Akt pathway renders tumour cells more resistant to Myc as an oncogenic driver, generating rare stem cell‐like cells that retain the original AT2 lineage, thereby overcoming the transformation bottleneck.^201^, ^202^ Interestingly, consistent relative up‐regulation of PI3K/AKT and MYC pathway genes was also observed in the trans‐differentiation from *EGFR* mutant LUAD to LUSC.^107^

### 5.2 Lineage imbalance: diverse developmental trajectories driving tumour progression

Tumour cells frequently forge novel developmental trajectories to expedite their progression. Yang et al.^48^ demonstrated that LUADs exhibit a pronounced propensity for gastrointestinal lineage transformation at an early stage. Little et al.^203^, ^204^ revealed that LUADs universally express gastrointestinal markers (PIGR/TFF2) following the early loss of spectral regulation by NKX2‐1. Coupled SOX2 overexpression in the context of NKX2‐1 deletion facilitates the formation of squamous carcinomas with oesophageal differentiation traits, though this is typically a transient stage or branch.^205^ In contrast, EMT is more prevalent in LUADs.

Lineage tracing studies have illustrated that LUAD cells originating from a common lineage progressively lose their alveolar characteristics during development.^48^, ^206^ This involves the depolymerisation of cell junctions, loss of polarity and the gradual accumulation of EMT pathway‐associated molecules. At this juncture, EMT is viewed as a formidable barrier in tumour therapy. Numerous factors, such as cytokines secreted by the tumour stroma, interactions between tumour cells and extracellular matrix components, Mechan‐transduction in the local environment and developmental signals from various sources within the microenvironment, drive EMT.^207^, ^208^, ^209^, ^210^ This process endows tumour cells with enhanced plasticity, facilitating their dissemination and colonisation in distant organs.

Nevertheless, most tumour cells do not undergo a complete EMT but instead deliberately adopt certain mesenchymal stem cell properties to metastasise while ensuring their cellular origin remains identifiable.^211^ For instance, the overexpression of ZEB, SNAIL and TWIST family factors promotes circulating tumour cell (CTC) release, VEGF‐A expression stimulates angiogenesis, and the production of proteolytic enzymes such as MMPs enhances tumour migration.^212^, ^213^ Studies indicate that LUADs, during the progression through pathological subtypes (lepidic, papillary, acinar and solid), not only lose their initial alveolar structure and molecular characteristics but also exhibit a molecular expression profile consistent with EMT.^63^ However, the transition from an epithelial to a mesenchymal‐like phenotype is not abrupt; it occurs in gradual steps, forming a gradient of metastable phenotypes wherein specific mesenchymal and epithelial traits coexist, ultimately culminating in a stable EMT program.^214^ Recent evidence suggests that cells in a hybrid EMT state, where epithelial and mesenchymal markers are co‐expressed, possess the highest malignant and metastatic potential.

## 6 SUMMARY OF THE RELATIONSHIP BETWEEN PLASTICITY AND LUNG CANCER DEVELOPMENT

Lineage plasticity dynamically regulates the differentiation states and phenotypic transitions of tumour cells, giving lung cancer cells a survival advantage when facing multiple physiological and therapeutic stresses. Studies in lung cancer models show that driver mutations are only oncogenic in specific lineage contexts or progenitor‐like states, suggesting that lineage regulation plays a decisive role in tumour initiation.

In the early stages of tumourigenesis, mutated cells typically do not directly exhibit a tumourigenic phenotype but exist in an intermediate ‘damage/plasticity’ state that resembles a normal lineage state. This state retains some normal cell characteristics while possessing potential for transformation, helping the cells adapt to the local metabolic environment after the driver mutation and reducing immune system recognition and clearance, thereby overcoming the initial ‘bottleneck’ in clonal expansion. However, lineage plasticity is not only involved in early clonal selection; as the tumour progresses, the initially dominant clones gradually decline due to changes in the microenvironment and resource depletion, while new clones or originally disadvantaged clones, induced by lineage plasticity, are positively selected for their distinct phenotypes and adaptive strategies. The tumour's lineage plasticity allows for this multi‐stage clonal competition and the maintenance of hybrid states, which are central mechanisms for lung cancer survival, evolution and resistance to treatment.

As tumour cells further escape initial lineage constraints, they evolve towards more plastic states through mechanisms such as TF regulation, epigenetic reprogramming and microenvironmental influences. In this process, cells can acquire new lineage identities, such as transitioning from adenocarcinoma to squamous carcinoma or small cell carcinoma, or undergoing EMT, finding a balance between adhesion and migration to adapt to oxidative stress, matrix mechanics and nutrient limitation in the new metastatic environment.

Finally, in response to targeted drugs, chemotherapy, radiotherapy and targeted therapy, lineage plasticity promotes the formation of multi‐clonal heterogeneity within the tumour. By dynamically altering cell states, inducing treatment escape‐associated TFs (such as SOX2, ASCL1), and activating pathways like PI3K/AKT and MYC, the tumour further enhances its ability to adapt to immune attack and metabolic stress. At the same time, subpopulations with different lineage backgrounds or rare ‘dormant’ progenitor‐like cell groups can withstand therapeutic invasion, subsequently remodelling the tumour structure and driving relapse. Therefore, lineage plasticity is not only the foundation for the origin and classification of lung cancer, but also underpins multiple stages of tumour evolution, metastasis and resistance. It gives tumour cells the ability to survive and adapt under various internal and external pressures. Understanding its dynamic changes is crucial for revealing the evolutionary paths of lung cancer and developing targeted therapeutic strategies.

## 7 RESEARCH PROGRESS OF TARGETED LINEAGE PLASTICITY

Current methodologies for continuous monitoring of solid tumours face substantial obstacles, making it challenging to fully grasp the transcriptomic landscape and the activation patterns of epigenetic factors driving lineage plasticity in patients. The swift progress in liquid biopsy technology, however, offers a promising avenue for the early identification and surveillance of lineage plasticity.

In lung cancer, CTC analysis can acutely detect heterogeneity in the expression of epithelial, EMT and stemness markers.^215^, ^216^ Research has particularly focused on SCLC due to its high proliferative capacity and the abundant presence of CTCs in the bloodstream.^217^ Moreover, it has been demonstrated that CTC detection is significantly more sensitive in advanced NSCLC.^218^ Cell‐free DNA (cfDNA) can capture a broad spectrum of tumour‐derived alterations, providing a non‐invasive view of clonal evolution and, indirectly, lineage plasticity. Assays typically survey driver mutations relevant to lung cancer biology and therapy resistance – such as KRAS, PIK3CA and EGFR – and clonal‐haematopoiesis (CH) genes like DNMT3A and TET2, which are monitored to distinguish tumour DNA from age‐related background variants.^219^, ^220^ While most of these mutations are not themselves lineage‐plasticity drivers, longitudinal cfDNA profiling of their rise or decline helps infer shifts in sub‐clone prevalence and, by extension, changing lineage states.

The analysis of DNA methylation status or other epigenetic marks is another highly promising approach. Numerous studies have confirmed the potential utility of cfDNA methylation profiling as a non‐invasive, cost‐effective, sensitive and accurate basis for early tumour detection.^221^ In a recent large‐scale clinical validation study, the Multi Cancer Detection Blood Test (MCDBT‐1/2) model, constructed from methylome information, exhibited high sensitivity, specificity and accuracy in predicting the origins of colorectal, oesophageal, liver, lung, ovarian and pancreatic cancers.^222^ Collectively, these programs can more robustly identify changes in lineage plasticity and other epigenetic drivers of disease progression.

Many clinical studies now employ liquid‐biopsy readouts – particularly circulating tumour DNA (ctDNA) – to guide treatment decisions in real time. By continuously tracking molecular‐marker dynamics, clinicians aim to match therapy more precisely to clonal evolution and lineage‐plasticity states, with the goals of improving efficacy, lowering toxicity and delaying resistance. Dong et al.^223^ conducted a non‐randomised single‐centre study (2020–2022, 60 patients with advanced NSCLC) in which *EGFR*‐TKI therapy was paused after local consolidative therapy whenever ctDNA was negative; treatment was restarted or stopped according to scheduled ctDNA/CEA monitoring. This ‘stop–start—withdraw’ strategy produced a median progression‐free survival (PFS) of 18.4 months, and up to 23% of patients required no additional therapy.^223^ The multi‐centre phase II/III trial CCTG BR.36 enrolled previously untreated advanced NSCLC patients (*EGFR/ALK*‐wild‐type, PD‐L1 ≥ 50%). Six weeks after starting treatment, ctDNA molecular response determined whether patients continued single‐agent immunotherapy or received added chemotherapy (platinum + pemetrexed/paclitaxel if ctDNA remained positive). Early data show high concordance between ctDNA response and radiographic response (sensitivity 82%, specificity 75%), correlating with better PFS and overall survival (OS).^224^, ^225^, ^226^ia et al.^227^ followed 233 patients with stage I–III NSCLC for 3 years after surgery, using serial ctDNA to detect minimal residual disease and to guide adjuvant therapy and recurrence surveillance. Small pilot studies have also explored daily urine‐ctDNA monitoring to gauge early response to OSI‐R‐TKI therapy (e.g., daily measurement of *EGFR*‐mutant ctDNA during osimertinib treatment).^228^

Collectively, these investigations embody the ‘dynamic‐gradient’ concept: therapy is adjusted according to sequential molecular monitoring, underscoring the importance of timing. Beyond gradient‐based strategies, many studies are also probing new targets and broad‐spectrum combination regimens.

New targets, such as DLL3 which is prominently expressed during NE transformation, is viewed as a promising target for SCLC treatment.^229^ Rovalpituzumab tesirine (SC16LD6.5), an antibody‐drug conjugate targeting DLL3, has shown promising results in phase I studies against SCLC and large‐cell NE tumours, demonstrating encouraging single‐agent anti‐tumour activity and a manageable safety profile. Although it was prematurely terminated in a subsequent study after failing to meet the intended midterm primary PFS and/or OS endpoints,^230^ other therapeutic regimens targeting DLL3, such as T‐cell engaging molecules (TCEs) and chimeric antigen receptor therapies, continue to show promising therapeutic potential.^231^ Tarlatamab (AMG 757), a bispecific T‐cell engager combining DLL3 and CD3, can induce T‐cell‐mediated tumour lysis, increase the number of effector T‐cells near the tumour and enhance anti‐tumour effects. Other TCEs and molecules targeting DLL3, including HPN328, BI 764532 and QLS31904, have entered phase I clinical trials and are being investigated in DLL3‐positive SCLC and other NE tumours.^232^, ^233^, ^234^, ^235^

Clinical trials involving broad‐spectrum multi‐target combinations have also garnered significant attention. The multi‐centre, randomised, open‐label phase III clinical trial IMpower150 evaluated the efficacy of atezolizumab (a PD‐L1 inhibitor) combined with chemotherapy (carboplatin + paclitaxel) and bevacizumab (an anti‐VEGF antibody) in patients with advanced NSCLC. The data demonstrated significant improvements in OS and PFS with this treatment regimen.^236^ The phase III randomised clinical trial CheckMate 9LA evaluated the combination of nivolumab (a PD‐1 inhibitor) and ipilimumab (a CTLA‐4 inhibitor) with chemotherapy in patients with advanced NSCLC, revealing that this combination therapy effectively improves immune evasion in the tumour microenvironment, significantly prolonging patient survival.^237^ The POSEIDON trial also suggested that in patients with advanced NSCLC, chemotherapy combined with PD‐L1 and CTLA‐4 inhibitors (triple combination: carboplatin + paclitaxel + durvalumab + tremelimumab) offers durable long‐term OS benefits compared with chemotherapy alone.^238^ Additionally, the combination of pembrolizumab and vorinostat (an HDACi) demonstrated good tolerability, and despite progression after prior immunotherapy, it still showed preliminary anti‐tumour effects.^239^

However, in clinical explorations of tumour dynamic monitoring and multi‐point combination therapy, there are still some contradictions and data that need to be interpreted with caution. In the multi‐centre ASSESS study in Europe and Japan, a total of 1162 tissue and plasma paired samples were included. The results showed that the specificity of ctDNA testing for *EGFR* mutations was 97%, with an overall concordance rate of about 89%, but the sensitivity was only 46%.^240^ Pender et al.^241^ retrospectively evaluated the *EGFR* ctDNA detection using the Cobas platform, finding a sensitivity of about 46% when compared with tissue control, and similar sensitivity was reported in the IGNITE study (46.9%). This result is also related to inconsistencies in the quantification methods between different studies, and therefore, relying solely on this detection result to abandon or adjust treatment plans is not recommended. Undoubtedly, dynamic ctDNA monitoring remains a powerful tool, but it should be used under strict monitoring and integration of multi‐source data, rather than as an independent criterion.

Combination therapies generally enhance efficacy but also bring higher toxicity risks. The POSEIDON trial, which evaluated durvalumab and tremelimumab combined with chemotherapy, showed improved efficacy. However, with prolonged treatment cycles, immune‐related adverse events (such as pneumonia and immune‐related lung diseases) gradually emerged, particularly in patients receiving high‐dose immunotherapy, where toxicity became difficult to control. In contrast, trials like IMpower133 also used immune combinations, but the reported treatment‐related toxicity was relatively lower, although efficacy was not consistently observed across all patient groups, especially in elderly patients or those with multiple comorbidities.^242^, ^243^, ^244^ The combination of Selinexor and Docetaxel in the phase I/II trial for *KRAS*‐mutant NSCLC showed some effect in *TP53* wild‐type patients but exhibited limited efficacy in the overall patient population, along with high treatment toxicity (such as neutropenia, gastrointestinal discomfort, etc.).^245^

Additionally, although multiple clinical trials have shown that immunotherapy combined with chemotherapy can improve efficacy, significant differences in the effect across different patient populations remain. The POSEIDON trial reported significant benefits with the combination of durvalumab and tremelimumab plus chemotherapy, especially in the high PD‐L1 expression group, where 5‐year OS data were favourable (significantly extended OS). However, in other trials combining immune therapy and chemotherapy, such as KEYNOTE‐407 and IMpower133, the treatment benefits in different patient groups were more limited, and the expected survival extension effect was not achieved. This may be due to differences in PD‐L1 expression levels among populations, and thus, PD‐L1 expression can help in selecting the appropriate treatment regimen.^242^, ^243^, ^244^ This may also explain the different responses observed in *KRAS*‐mutant patients treated with immunotherapy combined with chemotherapy.^246^

Additionally, in some clinical studies involving multi‐point combination therapies, the synergistic effects between drugs were not as pronounced as expected, and certain combination regimens did not significantly outperform monotherapy. The KEYNOTE‐598 trial showed that adding ipilimumab to the pembrolizumab regimen did not improve efficacy and, compared with using pembrolizumab alone as a first‐line treatment for metastatic NSCLC with PD‐L1 tumour proportion score ≥50% and no targetable *EGFR* or *ALK* abnormalities, it resulted in greater toxicity.^247^ However, the POSEIDON and CheckMate 9LA trials showed greater benefits for patients receiving combination therapy, which is not entirely contradictory to the results of KEYNOTE‐598. The main reasons for this discrepancy may lie in the differences in treatment regimens, immune microenvironment and patient population selection. POSEIDON and CheckMate 9LA employed a combination of immunotherapy and chemotherapy, which showed significant effects in patients with low PD‐L1 expression. In contrast, KEYNOTE‐598 mainly focused on the pembrolizumab + ipilimumab combination, and due to the absence of chemotherapy and the complexity of resistance, the results showed that combination therapy did not necessarily improve efficacy, but instead brought greater toxicity.

Multi‐point combination and dynamic gradient therapy holds great potential. Both historical and emerging evidence underscores the feasibility of targeting lineage plasticity in cancer treatment, especially with the support of personalised therapy and molecular monitoring. Epigenetic mediators such as EZH2, LSD1 and DNMT1 play pivotal roles in remodelling the epigenetic program and mediating immunosuppression.^74^, ^86^, ^87^ These studies suggest novel immunosensitising therapeutic strategies and herald new opportunities for tumour prevention and treatment through an enhanced understanding of lineage differentiation.^248^, ^249^ Future research will focus on issues such as subgroup differences in immunotherapy combinations, sensitivity limitations of ctDNA monitoring and the inconsistency of toxicity and efficacy in combination therapies. These contradictions and challenges indicate that more large‐sample, multi‐centre, personalised clinical trials are needed to further validate efficacy and safety, as well as to make precise treatment adjustments for different patient groups.

## 8 THE IMPACT OF LINEAGE PLASTICITY ON KEY CLINICAL OUTCOMES IN LUNG CANCER

Lineage plasticity plays a decisive role in multiple key clinical outcomes in lung cancer. First, during tumour initiation, driver mutations (such as *KRAS^G12D* or *EGFR* mutations) can only efficiently induce lesion formation in specific progenitor‐like or intermediate ‘damage/plasticity’ states. Otherwise, even if the cells carry the same mutations, they are unlikely to progress into tumours. This phenomenon explains why the oncogenic efficiency of the same driver mutations significantly differs in different cell lineage backgrounds and affects the differentiation degree and prognosis of early lesions. As the tumour progresses, the attenuation of lineage regulation leads to increased multiclonal heterogeneity – subpopulations maintaining epithelial characteristics coexist with subpopulations that have transitioned towards squamous or small‐cell phenotypes. This makes the tumour more prone to immune evasion and better able to adapt to therapeutic stress, ultimately leading to resistance, rapid disease progression and even histological transformation (e.g., *EGFR*‐mutant LUAD transforming to SCLC is often accompanied by *TP53/RB1* inactivation, significantly worsening prognosis).

Moreover, lineage plasticity dynamically regulates EMT and redifferentiation pathways, enabling tumour cells to acquire enhanced migration and invasion abilities, making them more likely to shed into circulation and survive as CTCs. They then reshape their lineage identity in distant organs, adapting to the new microenvironment and establishing metastatic foci. In this process, cells with a mixed phenotype retain adhesion capabilities while also exhibiting migratory features, greatly increasing the success rate of metastasis. At the same time, therapeutic stress (including targeted therapy, chemotherapy and radiotherapy) can induce tumour cells to enter a reversible ‘drug‐tolerant precursor’ state or DTP (drug‐tolerant persister) state. These cells temporarily evade drug‐induced killing by activating epigenetic reprogramming and stress response pathways (such as AKT/MYC, NF‐κB and antioxidant stress pathways). Once therapy is paused or the microenvironment changes, they can redifferentiate into multiple lineage branches, replenishing tumour heterogeneity and triggering disease relapse.

Thus, lineage plasticity not only determines the metastatic potential of tumour cells but also provides the biological basis for their dynamic escape and resistance evolution in the face of treatment. Understanding and targeting this plasticity mechanism, which spans tumour initiation, progression, transformation and resistance, is crucial for developing precise combination interventions (e.g., epigenetic inhibitors combined with *EGFR*‐TKI, ICI, AKT/MYC or DLL3‐targeted therapies) to improve clinical efficacy and prolong patient survival.

## 9 CHALLENGES AND FUTURE PERSPECTIVES IN LUNG CANCER LINEAGE PLASTICITY RESEARCH

There are still various limitations in the current research on lineage plasticity in lung cancer. First, differences in animal models limit the extrapolation of research findings to clinical settings. A series of studies comparing the structure and immune environment of mouse and human lungs have found significant differences: mouse lung development is faster and structurally simpler, lacking human‐specific RBs and certain progenitor cell populations; the immune system is also more ‘immature’ due to the absence of pathogen exposure. Moreover, mouse lung cancer models mainly produce tumours of a single subtype, making it difficult to replicate the histological heterogeneity and complex acquired resistance mechanisms seen in human lung cancer. These differences mean that the conclusions drawn from mouse models have limitations when extrapolated to humans, requiring cautious interpretation.

Second, limitations in sample sources lead to insufficient characterisation of the true tumour heterogeneity. Liquid biopsies, such as plasma cfDNA and CTCs, provide non‐invasive monitoring options, but their representativeness is still lacking and they cannot accurately reflect the complex heterogeneity within tissues.^250^ For example, current CTC detection methods do not achieve 100% sensitivity and specificity. Some tumour cells undergoing EMT may not be captured because they no longer express epithelial markers, and CTCs in clustered forms are more difficult to detect.^251^ cfDNA, on the other hand, is a mixture of DNA released from different tumour clones. In early‐stage disease, its concentration is very low and is mixed with a large amount of normal DNA background, which may dilute or omit certain subclonal signals.^250^ Thus, relying solely on liquid biopsy is insufficient to fully capture the spatial and evolutionary heterogeneity of solid tumours.

Additionally, there is insufficient understanding of the epigenetic information related to tumour lineage plasticity, and a lack of deep tracking of its dynamic changes. Most studies obtain ‘static’ snapshots of the genome or transcriptome at specific time points, lacking continuous monitoring of epigenetic markers during disease progression. For example, data on DNA methylation, histone modifications and chromatin accessibility are scattered and limited in patient samples, and we are still unable to map the complete picture of how epigenetic reprogramming during lineage transformation progresses over time. This lack of longitudinal tracking means that many critical moments may have been missed, limiting our understanding of the sequence and causality of plasticity events.

Last, the insufficient number and representativeness of samples limit the generalisability of conclusions, making it difficult to construct a comprehensive lineage evolution map. Many studies are based on small case numbers or single‐centre data, and the findings lack validation with larger sample sizes. Even conflicting results have been observed between different studies. This not only reflects the uncertainty of current evidence but also indicates that our overall understanding of lung cancer lineage evolution is still fragmented. It also suggests the need for larger‐scale, multi‐centre studies to integrate data from different subgroups and bridge the knowledge gap.

There are also blind spots and unresolved issues in the exploration of mechanisms. Although several key TFs and epigenetic regulatory molecules (such as SOX2, ASCL1, EZH2, LSD1, etc.) have been shown to play important roles in lineage conversion and resistance, the regulatory networks and induction mechanisms behind them remain unclear. The mechanisms of many lineage‐determining factors have not yet been elucidated, and how different signalling pathways coordinate to drive cell transitions across lineages remains controversial. For example, how microenvironmental factors such as hypoxia and inflammation interact with genetic or epigenetic mutations to promote lineage drift is still not well understood. Furthermore, basic questions such as which tumour cell subpopulations possess greater plastic potential and how this plastic state is maintained and reversed have not been definitively answered. These unknowns at the mechanistic level create blind spots in research, hindering our understanding of the full scope of lineage plasticity and its key components.

In clinical applications, there are still difficulties to overcome in the detection and intervention related to lung cancer lineage plasticity. On one hand, predicting tumour histological transformation and resistance evolution lacks accuracy. Clinically, tumour histological type conversion is often discovered only after the patient develops resistance or relapse, typically through biopsy. For example, *EGFR*‐mutant LUAD can transdifferentiate into small cell carcinoma, usually accompanied by TP53/RB1 inactivation and worsened prognosis. Due to the lack of biomarkers for early warning, physicians are often unable to identify these transformations in time, thus missing potential intervention opportunities. On the other hand, there is currently no routine method for real‐time dynamic monitoring of tumour lineage status. Imaging exams and sporadic tissue biopsies cannot capture the rapid lineage drift occurring inside the tumour, and although liquid biopsy technology holds promise, its sensitivity and consistency are still not ideal, with significant differences in detection results across studies. The ASSESS Study showed that plasma ctDNA testing for *EGFR* mutations had a sensitivity of only about 46%, suggesting that current liquid biopsy methods may miss a significant proportion of tumour changes. Furthermore, a standardised system of lineage markers has not yet been established. Although studies have explored NE markers, EMT‐related factors or specific gene mutation combinations as indicators of lineage conversion, no universally recognised marker has been applied in clinical monitoring.^252^ The lack of validated standards means that clinical decisions rely more on experience and post hoc test results, making it difficult to intervene in lineage plasticity changes in a timely and precise manner.

In summary, current research on lung cancer lineage plasticity has limitations at the levels of models, samples, mechanisms and clinical applications. These limitations not only restrict our in‐depth understanding of lung cancer biology but also affect the progress of translating lineage plasticity‐related knowledge into clinical benefits. Therefore, future research should prioritise the following directions: (1) Establish organoid and humanised models that more closely resemble human lung tissue structure and immune environments.^253^ Develop patient‐derived lung cancer organoids, tumour tissue‐immune cell co‐culture systems, and humanised mouse models to more accurately replicate the lineage evolution during lung cancer development. These new models would allow for simultaneous observation of tumour cell and microenvironment interactions, thereby improving the clinical relevance of mechanistic studies and compensating for the limitations of traditional mouse models. (2) Optimise liquid biopsy protocols to improve the sensitivity and specificity of ctDNA and CTC detection and integrate epigenetic information. Develop higher‐sensitivity, higher‐throughput detection technologies (such as digital PCR, single‐molecule sequencing and single‐cell analysis) to enhance detection capabilities for low‐abundance mutated clones and rare CTC populations. Additionally, incorporate epigenetic markers, such as cfDNA methylation profiles, into the analysis to more comprehensively capture the dynamic changes in tumour lineage states, providing a basis for real‐time monitoring and early warning. (3) Promote multi‐centre, multi‐time‐point longitudinal clinical sample collection and big data database construction.^250^ Further build large‐scale databases covering all stages of lung cancer, from early stages to metastasis and treatment. Systematically integrate samples from different patients across different time and spatial points. By tracking the lineage evolution of the same patient longitudinally and comparing the patterns across different patients, a panoramic map of lung cancer lineage plasticity can be drawn. Big data analysis may uncover reliable combinations of plasticity biomarkers and predictive models, offering decision support for personalised monitoring and intervention. (4) Conduct clinical trials on precise targeting of lineage plasticity with combination interventions. Introduce drug combination strategies targeting plasticity mechanisms alongside existing standard treatments and evaluate their efficacy in prospective trials. For example, combining epigenetic inhibitors (such as EZH2 or DNA methyltransferase inhibitors) with *EGFR*‐TKI or immunotherapy to inhibit the plasticity switch in tumour cells, delaying or preventing resistance and histological transformation. Alternatively, target specific overexpressed markers in lineage transformation (e.g., DLL3 in small cell transformation) for early intervention with targeted drugs or immunotherapy. Through these multi‐target combination therapy trials, we can verify whether inhibiting lineage plasticity improves patient prognosis and explore the best intervention timing and combination strategies.

## 10 CONCLUSION

In recent years, the concept of tumour lineage plasticity and its profound impact on tumour progression has garnered significant attention in the research community. Lung cancer exhibits pronounced lineage heterogeneity: distinct subtypes are shaped by specific driver genes and display unique phenotypic characteristics. Moreover, under selective pressures, tumour cells can even switch between lineage identities. This lineage plasticity spans every stage of lung cancer, from initiation through progression. This review consolidates the latest insights from the existing literature on the molecular mechanisms underpinning tumour lineage plasticity and its intricate interplay with the microenvironment. The findings underscore that tumour lineage plasticity is a pivotal mechanism by which cancer cells adapt to microenvironmental changes, evade immune surveillance and develop therapeutic resistance. We stress that lineage plasticity is not merely an adaptive response of late‐stage tumours to treatment pressure. Instead, it intervenes at the very inception of lung cancer, profoundly shaping the tumour's biological behaviour and evolutionary trajectory.

Tumour cell lineage plasticity is primarily orchestrated through epigenetic reprogramming, regulation by TFs and alterations in signalling pathways. For instance, TFs such as SOX2 and NKX2‐1 are crucial in mediating the transformation of LUAD to squamous carcinoma, while epigenetic regulators like EZH2 and LSD1 influence tumour cell differentiation and proliferation by modulating histone modifications and DNA methylation.^86^, ^87^, ^88^, ^89^ Furthermore, the hypoxic tumour microenvironment induces EMT via the activation of factors such as HIF‐1α, thereby enhancing tumour invasiveness and metastatic potential. Various components of the tumour microenvironment, including stromal cells, immune cells and secreted cytokines, significantly impact tumour lineage plasticity.^163^, ^164^, ^165^ Cytokines such as TGF‐β and VEGF not only govern tumour cell growth and differentiation but also indirectly facilitate tumour lineage plasticity by modulating stromal and immune cell functions.^135^, ^136^ Additionally, the mechanical properties of the tumour microenvironment, such as tissue stroma stiffness, can regulate the epigenetic landscape and transcriptional programs of tumour cells through the activation of the integrin–FAK–ERK signalling pathway.^254^

Targeting the key regulators of tumour lineage plasticity holds promise for developing innovative therapeutic strategies. For example, DLL3 has emerged as a critical therapeutic target for SCLC, with related antibody‐drug conjugates and bispecific T‐cell engagers demonstrating promising anti‐tumour activity in clinical trials.^198^ Furthermore, inhibitors of epigenetic modulators like EZH2, LSD1 and DNMT1 have shown potential in augmenting the efficacy of immunotherapy.^255^ Despite these advances, a comprehensive understanding of tumour lineage plasticity and its regulatory mechanisms remains elusive. Future research should delve deeper into the specific mechanisms of lineage plasticity across various tumour types and subtypes and elucidate their roles in tumour progression and treatment resistance. Concurrently, advancements in liquid biopsy technology are poised to enable real‐time monitoring of tumour lineage plasticity, thereby laying the foundation for personalised treatment approaches.

In conclusion, an in‐depth exploration of tumour lineage plasticity and its regulatory mechanisms is crucial for unveiling the core principles of tumour biology. Biomarkers of early lineage imbalance hold promise for predicting tumour behaviour and identifying high‐risk patients. Targeted interventions aimed at key pathways of lineage plasticity could delay or block tumour lineage transformation, overcoming treatment resistance and improving patient prognosis. Translating these mechanistic insights into new diagnostic and therapeutic strategies is a key direction for the future of precision medicine in lung cancer.

## AUTHOR CONTRIBUTIONS

Fanchen Meng: Writing – original draft and conceptualisation. Jianyu Li: Writing – review and editing and resources. Zhijun Xia: Writing – review and editing. Qinglin Wang: Writing – review and editing. Qinhong Sun: Writing – review and editing. Siwei Wang: Writing – review and editing. Lin Xu: Writing – review and editing and supervision. Rong Yin: Writing – review and editing and supervision.

## CONFLICT OF INTEREST STATEMENT

The authors declare no conflicts of interest.

## CONSENT

All authors reviewed and approved the manuscript.

## ETHICS STATEMENT

The authors have nothing to report.

## Data Availability

All data generated during this study are included in this published article.
